# Mitochondrial dysfunction impairs osteogenesis, increases osteoclast activity, and accelerates age related bone loss

**DOI:** 10.1038/s41598-020-68566-2

**Published:** 2020-07-15

**Authors:** Philip F. Dobson, Ella P. Dennis, Daniel Hipps, Amy Reeve, Alex Laude, Carla Bradshaw, Craig Stamp, Anna Smith, David J. Deehan, Doug M. Turnbull, Laura C. Greaves

**Affiliations:** 1 Wellcome Centre for Mitochondrial Research, Newcastle University, Newcastle upon Tyne, NE2 4HH UK; 2 Translational and Clinical Research Institute, Newcastle University, Newcastle upon Tyne, NE2 4HH UK; 3 Biosciences Institute, Newcastle University, Newcastle upon Tyne, NE2 4HH UK; 4 Bioimaging Unit, Medical School, FMS Professional Services, Newcastle University, Newcastle upon Tyne, NE2 4HH UK

**Keywords:** Mechanisms of disease, Bone

## Abstract

The pathogenesis of declining bone mineral density, a universal feature of ageing, is not fully understood. Somatic mitochondrial DNA (mtDNA) mutations accumulate with age in human tissues and mounting evidence suggests that they may be integral to the ageing process. To explore the potential effects of mtDNA mutations on bone biology, we compared bone microarchitecture and turnover in an ageing series of wild type mice with that of the *PolgA*^*mut/mut*^ mitochondrial DNA ‘mutator’ mouse. In vivo analyses showed an age-related loss of bone in both groups of mice; however, it was significantly accelerated in the *PolgA*^*mut/mut*^ mice. This accelerated rate of bone loss is associated with significantly reduced bone formation rate, reduced osteoblast population densities, increased osteoclast population densities, and mitochondrial respiratory chain deficiency in osteoblasts and osteoclasts in *PolgA*^*mut/mut*^ mice compared with wild-type mice. In vitro assays demonstrated severely impaired mineralised matrix formation and increased osteoclast resorption by *PolgA*^*mut/mut*^ cells. Finally, application of an exercise intervention to a subset of *PolgA*^*mut/mut*^ mice showed no effect on bone mass or mineralised matrix formation in vitro. Our data demonstrate that mitochondrial dysfunction, a universal feature of human ageing, impairs osteogenesis and is associated with accelerated bone loss.

## Introduction

Normal bone homeostasis requires maintenance of a delicate balance between the continuous processes of bone resorption by osteoclasts and new bone formation by osteoblasts^1,2^. Following attainment of peak bone mass in early adulthood, a decline in bone mineral density (BMD) ensues and continues unabated for the remainder of life in both humans and mice^3,4^. Progressive deterioration in bone microarchitecture with declining mineralisation levels and increasing porosity occurs, frequently culminating in osteoporosis^5–7^. The process affects males and females universally, with a transiently accelerated rate of loss observed in the latter secondary to menopausal oestrogen loss in humans^3,8^. Diminishing bone strength is inherent to declining BMD levels, the consequence of which is increasing risk of fragility fractures occurring with increasing age^9,10^. The pathogenesis of bone loss is not fully understood with various theories postulated such as increasing secretion of endogenous glucocorticoids^11,12^ and decreasing levels of sex hormones^13–16^, physical activity levels^17–19^ and insulin like growth factor I^20,21^. However, intracellular changes within bone tissue that occur with age, specifically accumulating mitochondrial DNA (mtDNA) mutations, may play a significant role in the failure of bone homeostasis leading to declining BMD levels.

With age, somatic mtDNA mutations accumulate in post mitotic tissues such a brain and muscle^22–25^, and mitotic tissue such as gut^26,27^. Mounting evidence in recent years, particularly that provided by animal models, has suggested that these may be intrinsic to the ageing process^28–30^. One of the most important functions of mitochondria in any cell is the production of energy in the form of adenosine triphosphate (ATP) via the process of oxidative phosphorylation^31^, although some ATP production can occur via much less efficient pathways such as glycolysis. For normal cellular function to occur, a minimum energy requirement must exist^32^. Cellular dysfunction has been shown to occur once genomes containing mtDNA mutations outnumber the presence of normal mtDNA sufficiently, depending on the type of mutations present, a phenomenon known as the threshold effect^33,34^.

mtDNA polymerase gamma (Polg) is the only DNA polymerase found in mitochondria and is responsible for replication and repair of mtDNA within all cell types^35^. The *PolgA*^*mut/mut*^ mitochondrial ‘mutator’ mouse possesses a defective version of the only proof reading domain of mtDNA polymerase^36^ which causes it to accumulate mitochondrial DNA point mutations at 3–5 times the rate of wild type mice resulting in a premature ageing phenotype, making it an excellent mouse model of ageing^37^. One of the most prominent consequences of this prematurely ageing phenotype is osteoporosis. Further evidence for mitochondrial dysfunction as a potential contributor to osteoporosis is seen in mice with a mitochondrial transcription factor A (TFAM) knockout specific to osteoclasts, the result of which is increased resorption when grown on dentine compared to normal osteoclasts^38^. Mice with global and osteocyte specific knockdown of superoxide dismutase (Sod2), an enzyme which protects against mitochondrial oxidative stress, also develop osteoporosis prematurely^39^.

Previous work studying the effects of exercise on *PolgA*^*mut/mut*^ mice, showed that regular exercise over a 5 month period ameliorated the increased rate of mtDNA mutations, increased mtDNA copy number and completely abolished the accelerated ageing phenotype, at 8 months of age. However, the effects of exercise on accelerated bone depletion was not evaluated^40^. Similarly, endurance exercise in human subjects has been shown to increase oxidative capacity of mitochondria within muscle, although the effects on bone mass are unknown^41,42^.

The normal function of osteoblasts in producing bone entails the production of a collagen framework and its subsequent mineralisation^43^. The susceptibility of this cell line to mtDNA mutations was previously unknown. We developed a quadruple immunofluorescence assay which accurately quantifies respiratory chain protein expression in bone cells, and demonstrated that mitochondrial COX-I and NDUFB8 protein expression in bone lining osteoblasts decreases with age in wild type mice (4 months vs. 11 months old), and is significantly reduced in *PolgA*^*mut/mut*^ mice aged 11 months compared to age matched wild type controls^44^. In order to evaluate the potential effects of osteoblast and osteoclast mitochondrial dysfunction on bone homeostasis, here we have assessed the effects of the *PolgA*^*mut/mut*^ genotype on bone phenotype, capacity of osteogenic cell lines to form mineralised matrix in vitro*,* osteoclast respiratory protein expression and resorption capacity in vitro*.* The effects of exercise on bone phenotype and in vitro mineralisation are also presented.

## Methods

### Mice

Mitochondrial mutator mice (*PolgA*^*mut/mut*^) were generated that had a knock-in missense mutation (D257A) in the second endonuclease proofreading domain of the *PolgA* catalytic subunit of the mtDNA polymerase, using a C57BL/6 background mouse^28^. Female and male mice were used, with comparisons made between *PolgA*^*mut/mut*^ and wild type controls from the previous generation to ensure no transmission of mtDNA mutations to ‘wild-type’ mice through the maternal line^45^. Animals were housed in single sex cages and cared for in compliance with the Animals (Scientific Procedures) Act 1984 in a purpose built facility. There was no variance in housing conditions, feed or water provision between *PolgA*^*mut/mut*^ and wild type littermate controls. All animal experiments were approved by and conducted in compliance with the UK Home Office (PPL P3052AD70) and the Newcastle University Animal Welfare Ethical Review Board (AWERB 425).

### Assessment of volumetric bone (BV/TV)

Lumbar spines and femurs were extracted from mice aged 4, 7 and 11 months and fixed for 72 h in 10% normal buffered formalin (NBF) after removal of soft tissues. Micro computed tomography (CT) scans of the lumbar spines and distal femur were then performed to determine BV/TV. Imaging was performed using a Skyscan 1,272 micro CT scanner. A beam intensity of 50Kv and 200µA was used with a 0.5 ml Al filter. A resolution of 4.3 µm and specimen rotation angle of 0.3° was used for femoral trabecular scans and a resolution of 8.6 µm and specimen rotation angle of 0.5 was used for femoral cortical scans. For lumbar vertebrae assessment, the L5 vertebrae was assessed, not including cortical bone or end plates. A resolution of 4.5 µm and a specimen rotation angle of 0.4 was used. Volumetric bone mass was recorded as a measure of bone volume (BV)/tissue volume (TV). Trabecular thickness, trabecular separation and trabecular number were also recorded in femoral and lumbar vertebrae trabecular bone. Cortical thickness was measured in diaphyseal femoral cortical bone.

### Bone histomorphometry

Tibiae from female mice aged 7 months were dissected, fixed in 4% formaldehyde for 24 h and embedded in methyl methacrylate without decalcification. 5 μm sections were taken for bone histomorphometry, performed as detailed previously^46^. In brief, sections were stained with Von Kossa stain to analyse bone mineralisation, 1% Toluidine blue to analyse osteoblast number, and stained for tartrate-resistant acid phosphatase (TRAcP) to analyse osteoclast number and surface area. To study the dynamic properties of bone, mice were injected intra-peritoneally 10-days and 2-days prior to sacrifice with 30 mg/kg alizarin complexone and 20 mg/kg calcein in a 2% sodium bicarbonate pH 7.4 solution, respectively. Trabecular bone histomorphometric measurements were taken from fluorescent images of 5 μm sections acquired using a Zeiss Axio Imager 2 microscope and were used to calculate the bone formation rate (BFR). All bone histomorphometry analyses were performed on matched sections from 3 mice per genotype and were performed on the trabecular bone in the proximal tibia, 100 μm below the physis.

### In vitro assessment of mineralised matrix formation by osteogenic cells

Cells from mice aged 4, 7 and 11 months were compared. Following dissection of mouse femurs, the epiphyses were cut and the bone marrow from individual femurs was harvested by flushing the intramedullary canal with DMEM using a 25G needle. Each sample was centrifuged at 300× *g* and the supernatant removed, before the cell pellet was suspended in a T25 flask in 5mls of alpha modified minimum essential media (MEM). Alpha MEM was supplemented with 10% FBS, 2 mM L-glutamine, penicillin (100 u/ml), streptomycin (100 µg/ml) and amphotericin (0.25 µg/ml). After 24 h of incubation at 37 °C non-adherent cells were removed, aided by PBS wash steps, following which fresh media was applied. Media was changed again on day 6 and cells were harvested for use in mineralisation assays on day 10. At this point, cells were washed with PBS and dissociated using TrypLE express. Cells from each mouse were then plated at a density of 6.5 × 10^4^ in all wells of a 12 well plate in osteogenic media (alpha MEM supplemented as above with the addition of 50 µg/ml sodium ascorbate and 2 mM beta-glycerophosphate^47^. A full media change was performed every 3 days for 21 days at which point the cells were fixed briefly in 4% PFA. 7 of the wells were washed in 70% ethanol, air dried, stained with 2% alizarin red (which binds to calcium within mineralised bone) in dH_2_O for 10 min, washed 3 times with 50% ethanol and air dried. The 5 remaining wells were washed with TBS prior to ALP staining which was performed by combining 10 mg napthol MX (dissolved in 400 µl N,N-Dimethylformamide), with 60 mg of fast blue RR salt (Sigma F0500) in 100mls of tris-buffered saline (TBS), at a pH of 8.2. Cells were incubated in the dark for 30 min in this solution, following which, nuclei of cells within the same 5 wells were stained with Hoechst. As per previous work, we confirmed osteogenic differentiation in vitro by staining for ALP activity^47–53^. ALP is a phospho-ester, imperative to the mineralisation process^54,55^.

Fluorescent microscopy (405 nm) was used to image Hoechst stained cells, with 15 images taken from each of 5 wells at 4× magnification. Matlab automated software was used to record cell counts from these images. 26 colour brightfield images were taken of all alizarin red and ALP stained wells at 4× magnification. Image analysis was performed using an RGB filter within Volocity (PerkinElmer, Coventry, UK) software to detect alizarin red staining and the purple of ALP staining. The average surface area of mineralisation as depicted by alizarin red staining was recorded for each mouse cell line, as was the surface area and number of osteogenic cells, identified by positive ALP staining. Final results were expressed as average surface area of mineralisation per average area of ALP stained cells. To calculate population density of osteogenic cells in each image, the ratio of ALP positive cells to total cell count per image (as identified by Hoescht nuclear staining) was also recorded for each cell line.

### Effects of exercise on bone and phenotype and in vitro mineralisation

A subset of male *PolgA*^*mut/mut*^ mice were exercised from the age of 16 weeks, for 4 days per week using a treadmill. Following an initial 10-week acclimatisation period of gradually increasing intensity, the final protocol consisted of the mice running at 12 cm/s for 5 min, 20 cm/s for 40 min, and finally for 12 cm/s for 5 min on each of the 4 days. Tissue was harvested from mice aged 11 months to study whether exercise had any effects on bone density and functional capacity of osteogenic cells, in comparison to non-exercised *PolgA*^*mut/mut*^ mice aged 11 months.

### In vitro assessment of osteoclast function

Osteoclasts from male *PolgA*^*mut/mut*^ and wild type litter mates, aged 11 months were compared using a previously published method^56^. On day 1, following dissection of mouse femurs, the epiphyses were cut and the bone marrow from individual femurs was harvested by flushing the intramedullary canal with DMEM using a 25G needle. Each sample was centrifuged at 300× *g* and the supernatant removed, before the cell pellet was suspended in a T25 flask in 5mls of alpha modified minimum essential media (MEM). Alpha-MEM was supplemented with 10% FBS, 2 mM L-glutamine, penicillin (100u/ml), streptomycin (100 µg/ml) and amphotericin (0.25 µg/ml), 10^−7^ M prostaglandin E_2_ and 2.5 ng/ml of M-CSF. Cells were incubated for 24 h at 37 °C in an incubator supplemented with 5% CO_2_.

On day 2, non-adherent cells from each flask were removed, aided by PBS wash steps. These were centrifuged at 300× *g* for 5 min and the supernatant removed. The cell pellet was then suspended in alpha MEM at a density of 5 × 10^6 ^cells/ml. Alpha-MEM was supplemented at this stage and for the remainder of the experiment with 10% FBS, 2 mM L-glutamine, penicillin (100 u/ml), streptomycin (100 µg/ml), amphotericin (0.25 µg/ml), 10^-7^ M prostaglandin E_2_, 10 ng/ml M-CSF and 3 ng/ml of mouse RANKL. Using 96-well plates, cells from each mouse were seeded on to four 0.5 mm dentine discs (1 × 10^6 ^cells per disc in 200 µl media. Dentine was derived from walrus tusk (Oxford Biosystems), and had been pre-soaked in PBS.

Following a further 24 h, on day 3, discs were transferred to 6 well plates, with 2 mls of fresh supplemented alpha MEM in each well. A 50% media change was carried out on day 5. On day 7, a full media change was performed with the media having been acidified with the addition of HCl to reduce the pH to 6.9 (at incubator conditions) to activate osteoclast resorption. After 24 h in acidified media, dentine discs were washed in PBS and fixed using 2% glutaraldehyde in Sorensen’s solution, for 5 min.

TRAP staining was performed to detect osteoclasts. Osteoclasts were defined as TRAP positive, multi-nucleated cells. TRAP staining solution, pH 5.0, contained 0.1 M sodium acetate, 7.5 mM L-( +) tartaric acid, 0.2 mM naphthol AS-MX and 1.5 mM fast red violet LB salt (Sigma Aldrich). Cells were stained at 37 °C for 30 min. Transmitted light microscopy at 10× optical magnification was used to record images from 10 different areas of each disc. NIS-elements advanced research software (Nikon) was used to identify multinucleated TRAP positive osteoclasts to derive an average number of cells per mm^2^.

Following osteoclast cell counts, cells were removed from the dentine discs using cotton buds and 0.25 M ammonium hydroxide. The dentine discs were then stained by immersion in 1% toluidine blue dissolved in 0.1 M sodium tetraborate for 3 min. Discs were then rinsed in water to remove excess staining. Transmitted light microscopy was used at 10 × optical magnification to take 10 images from different areas of each disc. Using NIS-elements advanced research software (Nikon), average values were obtained for number of pits/mm^2^ and average pit size (µm) for each disc.

### In vivo assessment of osteoblast and osteoclast mitochondrial respiratory chain protein expression

Quadruple immunofluorescence was performed to quantify levels of mitochondrial proteins in osteoblasts and osteoclasts as previously described^44^. Comparisons were made between wild type mice aged 4 and 11 months, and *PolgA*^*mut/mut*^ mice aged 11 months. Data from 4-month-old wild type animals were used as the reference control. An antibody to cathepsin K was used to detect osteoclasts, and respiratory chain complex subunits within were detected using mouse monoclonal primary antibodies against NADH dehydrogenase [ubiquinone] I beta subcomplex subunit 8 (NDUFB8) and cytochrome *c *oxidase (COX) subunit 1 (COXI). These were applied in combination with a monoclonal antibody against the outer mitochondrial membrane protein porin (VDAC1). Porin is a nuclear-encoded, voltage gated ion channel present in abundance in the mitochondrial membrane, and its presence serves as a marker for mitochondrial mass^57^. NDUFB8 is a nuclear-DNA encoded subunit of Complex I^58^ and COX-I is a mtDNA-encoded subunit of Complex IV^59^.

Imaris image analysis software (Bitplane, v.8.4) was used to detect osteoclasts (546 nm) and areas within these that were positive for the mitochondrial mass marker porin (405 nm). For each of these areas of intracellular mitochondrial staining, the software provided average signal intensity values for porin (405 nm), COX-I/MTCO1 (488 nm) and Complex I/NDUFB8 (647 nm) within each cell.

### Statistical analysis

Prism (GraphPad) was used for all statistical analysis of micro CT scan, histomorphometry and cell culture data. Rstudio was used for analysis of osteoclast immunofluorescence data.

## Results

### Micro-CT scanning of femurs and lumbar spines demonstrates accelerated bone loss in *PolgA*^*mut/mut*^ mice

**Figure 1:**
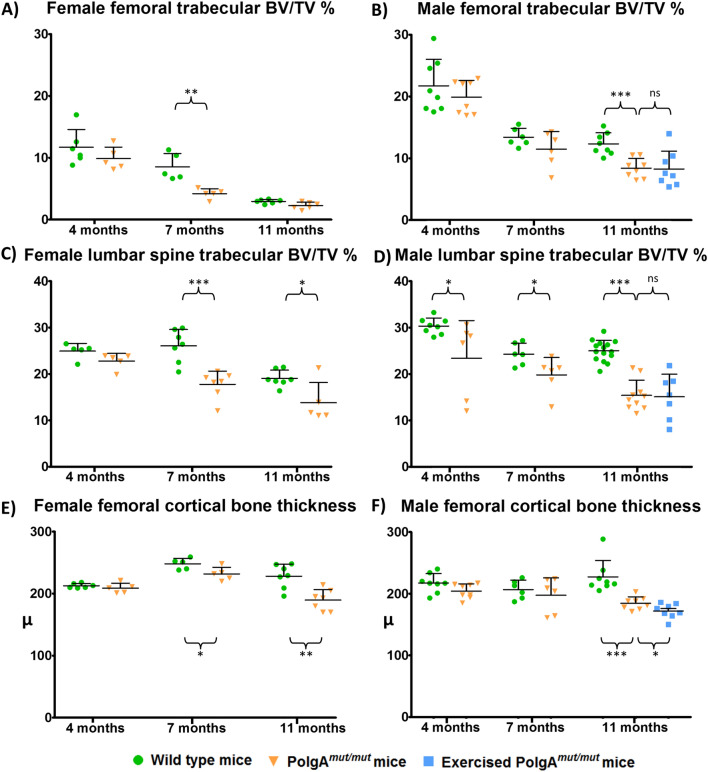
Micro-CT scan of femurs and lumbar spines demonstrate accelerated bone loss in *PolgA*^*mut/mut*^ mice. An exercise intervention was not associated with a change in trabecular bone mass. A significant decline in femoral trabecular BV/TV can be seen by 7 months of age in female (**A**) *PolgA*^*mut/mut*^ mice (*p* = 0.003) and by 11 months of age in male (**B**) *PolgA*^*mut/mut*^ mice (*p* < 0.0001) when compared to age matched wild type controls (unpaired 2 tailed t-test). A similar pattern of bone loss was seen in the lumbar spine with significant reductions in trabecular BV/TV in female *PolgA*^*mut/mut*^ mice at 7 and 11 months (**C**), and in male *PolgA*^*mut/mut*^ mice at 4, 7 and 11 months (**D**), compared to age and sex matched wild type controls. Cortical thickness was also significantly reduced in female *PolgA*^*mut/mut*^ mice at 7 and 11 months (**E**), and in male *PolgA*^*mut/mut*^ mice at 11 months (**F**), compared to wild type controls. Exercise had no significant effects (ns) on bone mass observed in 11 month old *PolgA*^*mut/mut*^ males in terms of trabecular BV/TV (**B**, **D**). A gradual decline in BV/TV levels is observed in wild type mice as a feature of advancing age, with females showing a significantly reduced femoral trabecular BV/TV in comparison to age matched males at 4, 7 and 11 months of age (*p* = 0.0004, *p* = 0.0015, *p* < 0.0001 respectively (unpaired 2 tailed t-test)).

Bone volume/tissue volume (BV/TV) of extracted femurs was evaluated using micro-CT scanning (Fig. 1) and unpaired 2-tailed t tests for comparisons. The femoral BV/TV decreased at an accelerated rate in *PolgA*^*mut/mut*^ mice compared to age and sex matched wild type controls with a significantly reduced trabecular BV/TV in both female and male *PolgA*^*mut/mut*^ mice by the ages of 7 and 11 months respectively (*p* = 0.003 and < 0.0001). The same comparisons (Supplemental Data S1) also show reduced levels of trabecular bone thickness, increased trabecular separation and reduced trabecular number in 11 month old *PolgA*^*mut/mut*^ males (*p* = 0.0009, 0.007 and 0.025 respectively) and in 7 month old *PolgA*^*mut/mut*^ females (*p* = 0.251, < 0.0001 and 0.004 respectively). A decrease in trabecular BV/TV of male and female wild type mice, with increasing age is also observed. Female wild type mice demonstrate significantly reduced BV/TV compared to age matched wild type males at all 3 ages studied (*p* < 0.001).

Assessment of cortical bone thickness (Fig. 1 and Supplemental Data S1) showed that it continued to increase in wild type mice between the ages of 4 and 7 months in females, in keeping with previous work^40^. However, significantly reduced cortical thickness was seen in *PolgA*^*mut/mut*^ female mice at 7 months (*p* = 0.032), and at 11 months of age (*p* = 0.005). Male *PolgA*^*mut/mut*^ mice exhibited significantly reduced cortical thickness at 11 months of age (*p* = 0.0008).

Trabecular bone from extracted lumbar vertebrae (L5) was also assessed (Fig. 1 and Supplemental Data S2). Compared with wild-type controls, a significantly reduced BV/TV was observed in *PolgA*^*mut/mut*^ males at 4, 7 and 11 months of age (*p* = 0.035, 0.033, and < 0.0001 respectively), and in *PolgA*^*mut/mut*^ female mice at 7 and 11 months of age (*p* = 0.0004 and 0.007 respectively). This corresponded with reduced trabecular thickness at 7 and 11 months in *PolgA*^*mut/mut*^ males (*p* = 0.016 and 0.002 respectively) and *PolgA*^*mut/mut*^ females (*p* = 0.040 and 0.0001 respectively). In *PolgA*^*mut/mut*^ males, increased trabecular separation was observed at 4, 7 and 11 months (*p* = 0.005, 0.027 and 0.003 respectively), with reduced trabecular number at 4 and 11 months (*p* = 0.033 and < 0.0001 respectively). *PolgA*^*mut/mut*^ females exhibited increased trabecular separation and reduced trabecular number at 7 months (*p* = 0.013 and < 0.0001 respectively). Again, exercise was not associated with any difference in trabecular bone mass observed in *PolgA*^*mut/mut*^ male mice at 11 months.

Comparison of exercised and non-exercised male *PolgA*^*mut/mut*^ mice aged 11 months demonstrated that exercise did not affect any of the studied parameters on micro CT scan of whole femurs (trabecular BV/TV, trabecular thickness, trabecular separation, trabecular number) or lumbar vertebrae (trabecular BV/TV, trabecular thickness, trabecular separation, trabecular number). However, a reduction in cortical thickness was observed in exercised *PolgA*^*mut/mut*^ mice (*p* = 0.038).

### Histomorphometry shows reduced BV/TV, reduced bone formation rate, reduced osteoblast population density, and increased osteoclast population density in *PolgA*^*mut/mut*^ mice

The extracted tibiae of age matched wild type and *PolgA*^*mut/mut*^ mice were compared (unpaired 2 tailed t-tests). BV/TV and mineralisation (osteoid surface/bone surface) were found to be significantly lower in *PolgA*^*mut/mut*^ mice (*p* = 0.017 and 0.010 respectively) compared to age matched wild type mice.

**Figure 2:**
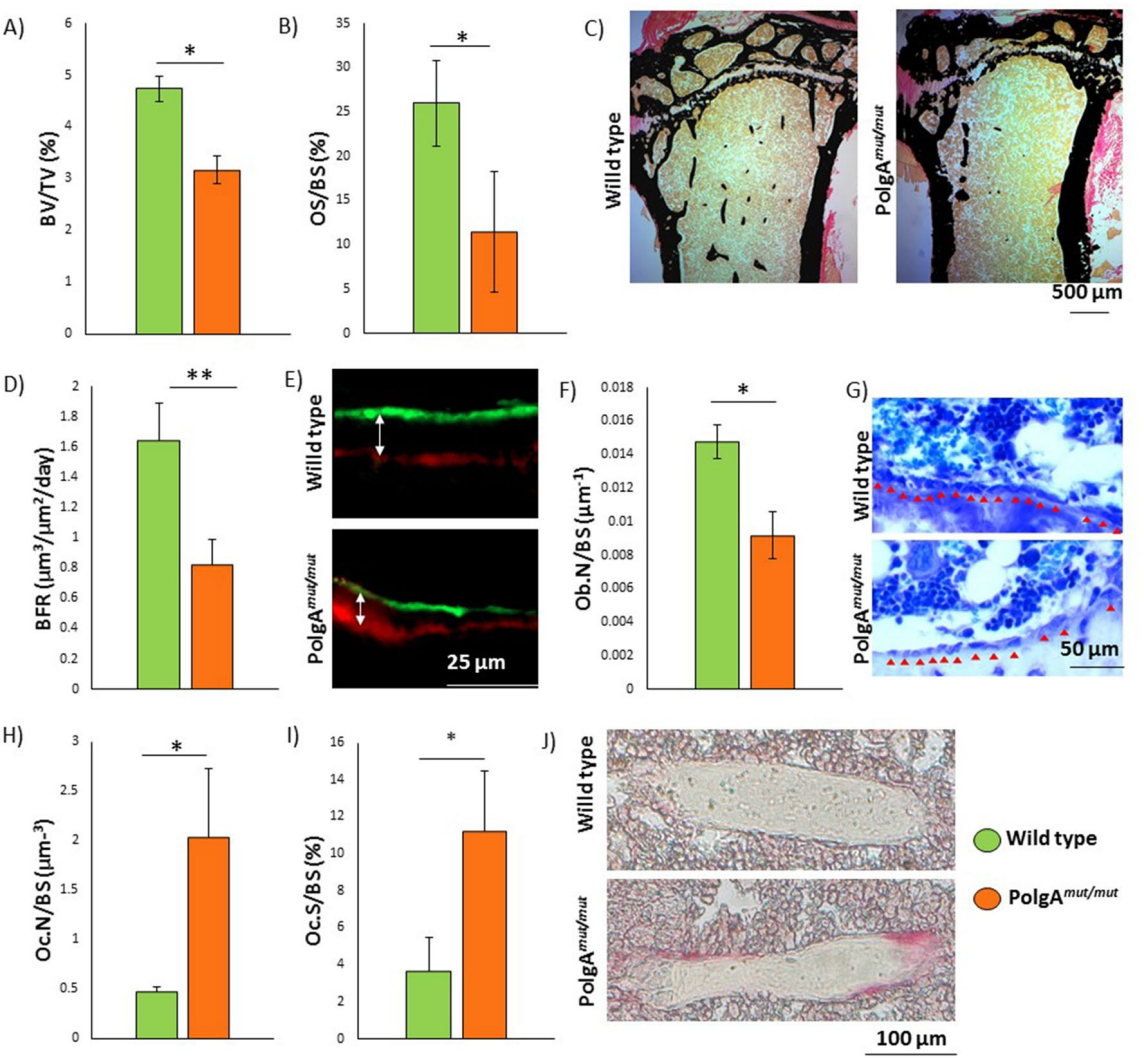
Hitomorphometric analysis shows reduced bone density, reduced bone formation rate, reduced osteoblast population density, and increased osteoclast population density in *PolgA*^*mut/mut*^ mice. BV/TV (**A**) and mineralisation (OS/BS%) (**B**) is significantly lower in *PolgA*^*mut/mut*^ mice compared to age matched wild type (WT) mice (*p* = 0.017 and 0.010 respectively). von Kossa staining of WT and *PolgA*^*mut/mut*^ mice (**C**). BFR were significantly lower (*p* = 0.0088) in *PolgA*^*mut/mut*^ mice compared to age matched WT (**D**). Immunofluorescent imaging of tissue sections from tibiae of WT and *PolgA*^*mut/mut*^ demonstrate the typical difference in BFR observed between the two genotypes (**E**). Significantly reduced osteoblast population densities (*p* = 0.005) in *PolgA*^*mut/mut*^ mice compared to age matched WT mice are observed (**F**). Typical appearances of WT and *PolgA*^*mut/mut*^ tibiae sections stained with toluidine blue, with red arrows demarcating bone-lining osteoblasts (**G**). There are significantly increased population densities of osteoclasts in *PolgA*^*mut/mut*^ mice compared to age matched WT, with higher osteoclast numbers (**H**) and osteoclast surface area relative to bone surface (**I**) (*p* = 0.017 and 0.023 respectively). Typical appearance of TRAcP stained tibiae sections from WT and *PolgA*^*mut/mut*^ mice (**J**).

Analysis of mineralisation fronts following staged injection of alizarin complexone and calcein demonstrated significantly reduced bone formation rates (*p* = 0.0088) in *PolgA*^*mut/mut*^ mice. The in vivo population densities of osteoblasts in *PolgA*^*mut/mut*^ mice was found to be significantly lower (*p* = 0.005), but conversely, that of osteoclasts was significantly (*p* = 0.017) higher (Fig. 2).

### Functional capacity of osteogenic cells to produce mineralised matrix in vitro is reduced in *PolgA*^*mut/mut*^ cells

**Figure 3:**
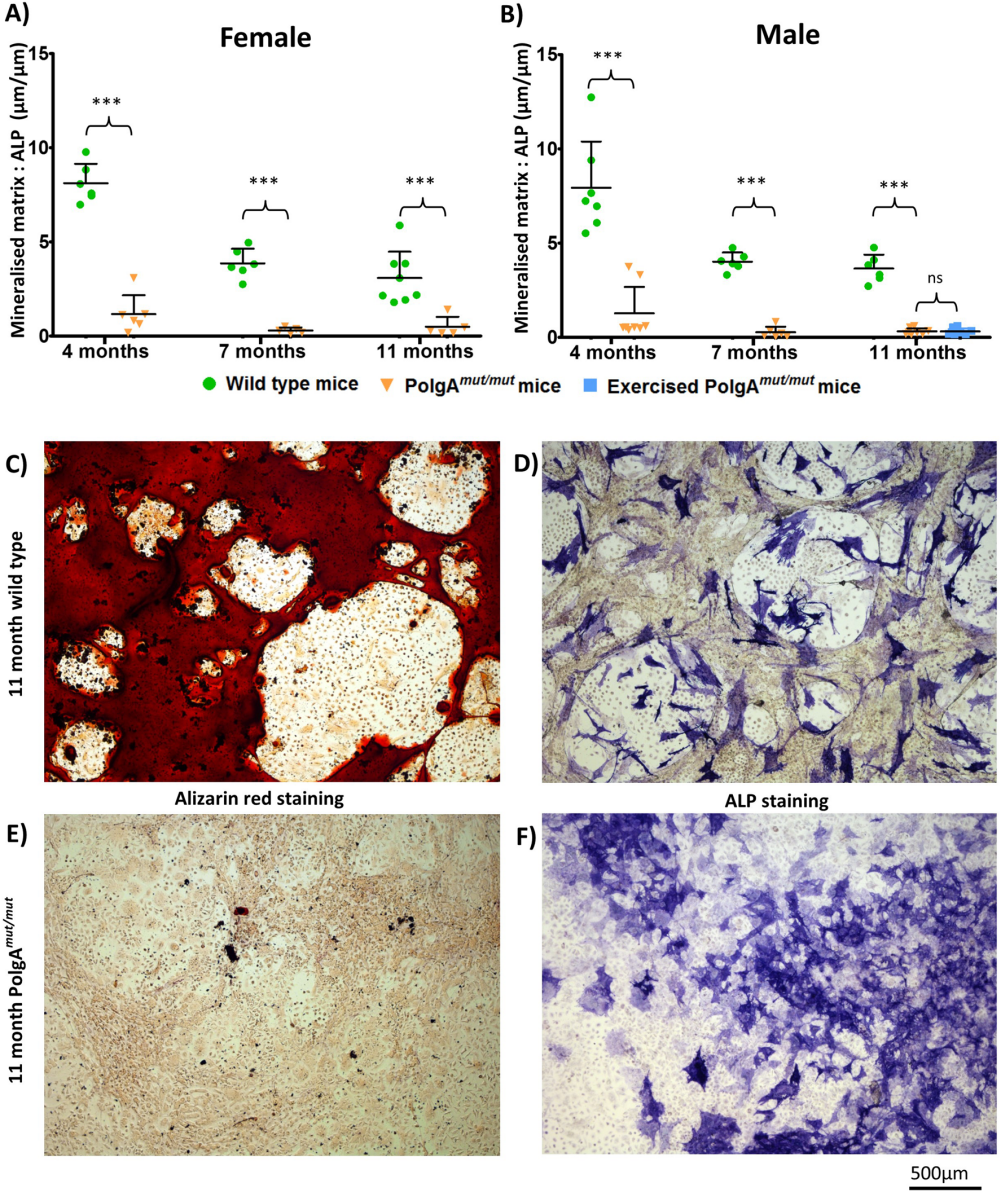
*PolgA*^*mut/mut*^ osteogenic cells produce less mineralised matrix in vitro*.* Ratio of mineralised matrix surface area to surface area occupied by osteogenic ALP positive cells. A gradual decline in mineralised matrix formation is seen with increasing age in wild type cells extracted from both females (**A**) and males (**B**), with a significant reduction observed by 7 and 11 months in comparison to cells from mice aged 4 months (*p* < 0.001, one way ANOVA, Bonferroni). At all three ages of study, and in both sexes, mineralisation by *PolgA*^*mut/mut*^ cells is vastly reduced (*p* < 0.0001, unpaired, 2 tail t-testing). The functional capacity of *PolgA*^*mut/mut*^ cells from 11-month old exercised male mice to produce mineralised matrix was not significantly (ns) different to that observed in cells derived from non-exercised 11 month old male *PolgA*^*mut/mut*^ mice (*p* = 1). Typical appearances of wild type cell lines (**C**, **D**) and *PolgA*^*mut/mut*^ cell lines (**E**, **F**) taken from mice aged 11 months are shown.

Mineralisation assays were performed using bulk unfractionated skeletal mesenchymal cells. The average surface area of mineralised matrix formed and corresponding surface area occupied by cells expressing ALP were recorded (Supplemental Data S3) for each cell line. Wild type osteogenic cells consistently produced mineralised matrix in abundance as demonstrated by increased alizarin red staining. The ratio of mineralised matrix surface area to surface area occupied by ALP positive cells (Fig. 3) fell with increasing age in the wild type mice, with significant reduction in mineralised matrix formation by 7 and 11 months in comparison to that at 4 months in both sexes (one way ANOVA, Bonferroni *p* < 0.001). Osteogenic cells differentiated from cells harvested from male and female *PolgA*^*mut/mut*^ mice aged 4, 7 and 11 months all produced significantly less mineralised matrix in comparison to age and sex matched wild type controls (*p* < 0.0001, unpaired 2 tailed t-test).

The exercise intervention made no significant difference to the capacity of osteogenic cells extracted from *PolgA*^*mut/mut*^ mice aged 11 months to produce mineralised matrix in vitro*.* No significant difference was observed in propensity for cell differentiation to ALP expressing osteogenic cells in this cell line either.

### Propensity of osteogenic cell differentiation to cells expressing ALP is unaffected in MSCs harvested from *PolgA*^*mut/mut*^ mice

**Figure 4:**
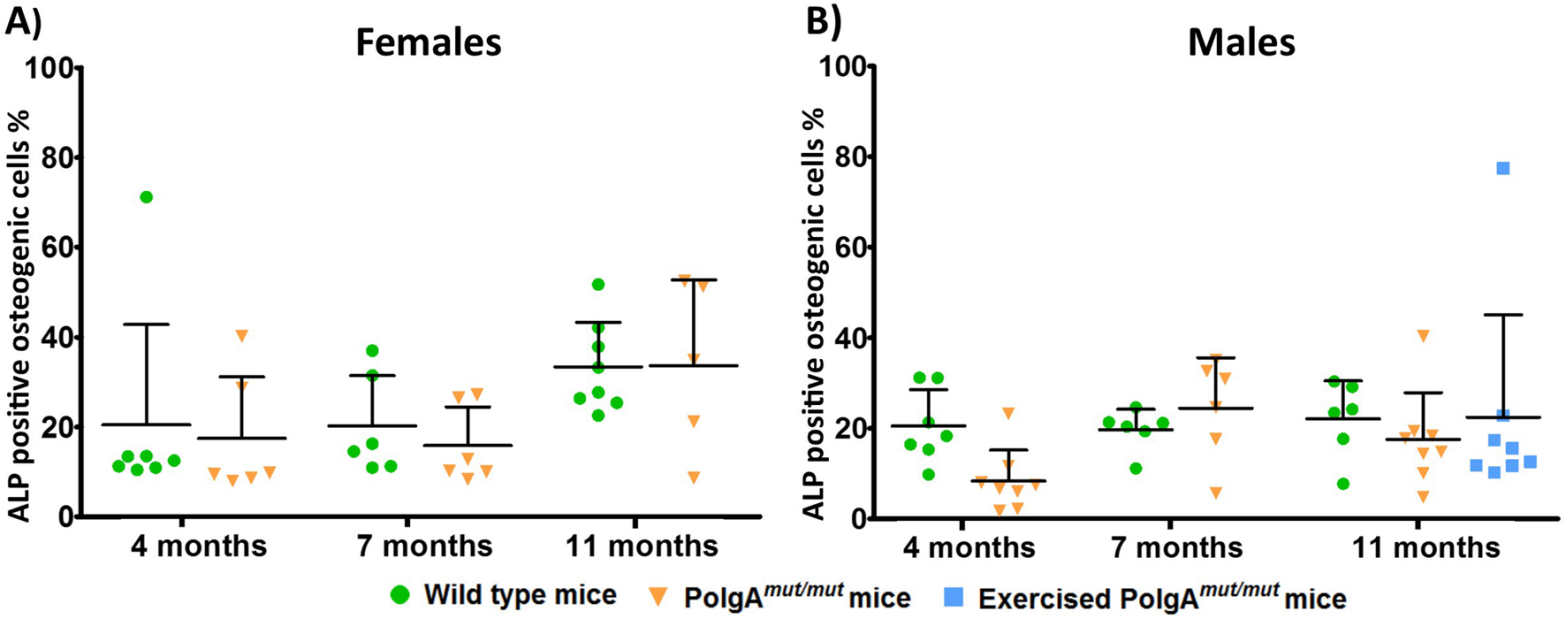
Population density of ALP positive, osteogenic cells, derived from bulk unfractionated mesenchymal cell harvests. The ratio of ALP positive cells to total cell number was recorded as a measure of osteogenic cell population density, for female (**A**) and male (**B**) mice. Significant variation in the population density of ALP positive cells occurred in all groups. There was no significant difference (one-way ANOVA/Bonferroni) between wild type cells and *PolgA*^*mut/mut*^ cells (regardless of sex or age of mouse of origin), in their propensity to produce osteogenic cells, suggesting that reduced mineralised matrix formation in vitro by *PolgA*^*mut/mut*^ cell lines is not accounted for by reduced population density of osteogenic cells.

The population density of ALP positive cells derived from bulk unfractionated skeletal mesenchymal cell harvest was examined by correlating the ratio of number of ALP positive cells to the number of all cell types (identified by nuclear Hoescht staining) for each mouse of origin (Fig. 4). One-way ANOVA and Bonferroni post-test comparisons showed no significant difference between wild type and *PolgA*^*mut/mut*^ cell lines in their propensity for osteogenic cell differentiation in male and female cell lines at all 3 ages. Significant variation in final population density of ALP positive cells is evident in age/sex matched groups with no significant positive correlation (Pearson) found in any age/sex matched cell line between the number of ALP positive cells formed and total number of cells present as identified by Hoechst nuclear staining (Supplemental Data S4).

### Osteoclast in vitro resorption assays demonstrate increased osteoclast activity

Following monocyte extraction from mice aged 11 months, and osteoclast culture on dentine discs, cells were removed and resorption pits were studied. Unpaired 2 tailed t-tests were performed for all comparisons. The resorption pits formed by wild type osteoclasts were larger than those formed by *PolgA*^*mut/mut*^ osteoclasts with average sizes of 534 µm and 333 µm respectively (*p* = 0.0008).

**Figure 5:**
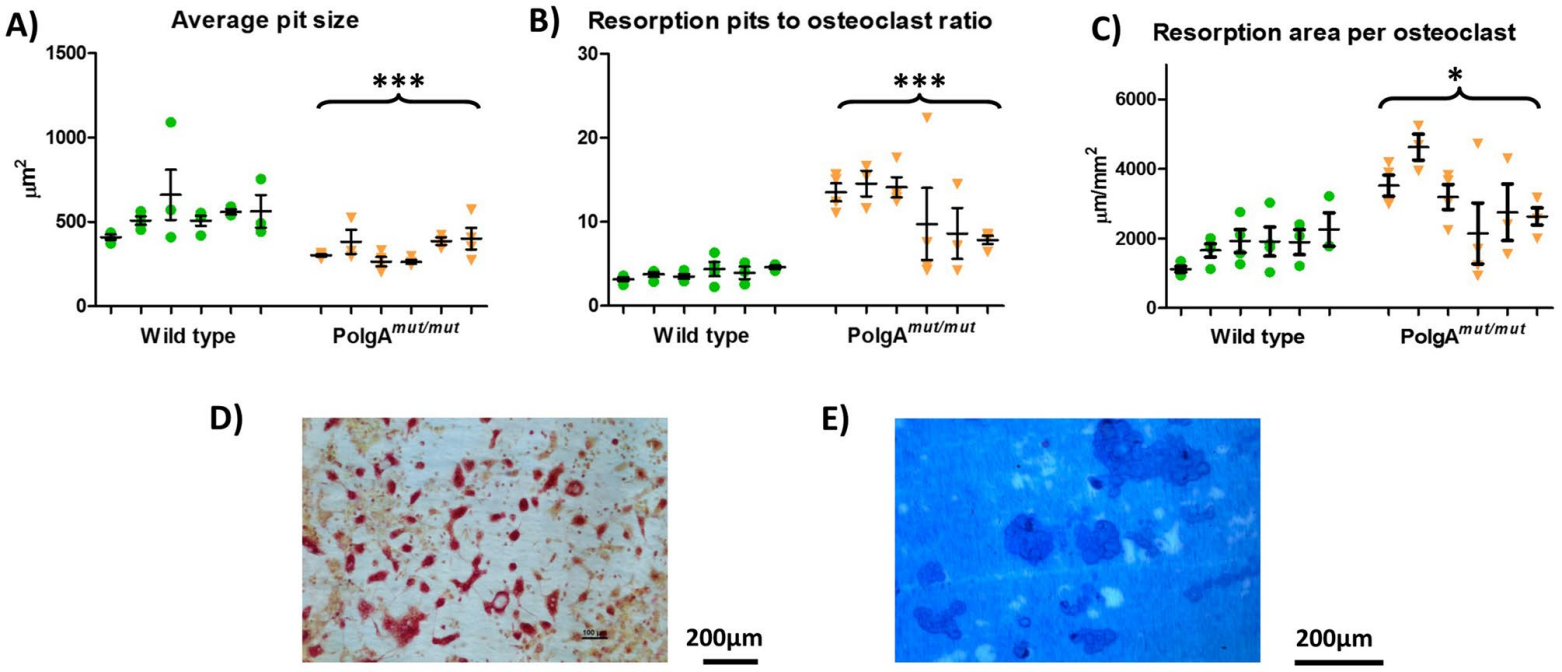
Osteoclast resorption assays using cells extracted from wild type and *PolgA*^*mut/mut*^ mice aged 11 months. Comparisons between wild type (WT) and *PolgA*^*mut/mut*^ cell lines. Average size of resorption pits formed by WT osteoclasts (**A**) was larger than those formed by *PolgA*^*mut/mut*^ osteoclasts (*p* = 0.0008). The ratio of resorption pits to osteoclasts was higher (**B**) in *PolgA*^*mut/mut*^ cells (*p* < 0.0001). The ratio of resorbed dentine to osteoclast number (**C**) was also higher in *PolgA*^*mut/mut*^ cells (*p* = 0.0134). Examples of appearances of osteoclasts on dentine (**D**) and visualisation of resorption pits (**E**).

The ratio of resorption pits to osteoclasts was analysed (Fig. 5). The ratio of pits to osteoclasts was significantly higher following *PolgA*^*mut/mut*^ osteoclast culture, with 9.3 pits observed per osteoclast compared to only 3.2 pits per osteoclast following wild type osteoclast culture (*p* < 0.0001). The total resorption area per osteoclast was also significantly higher following *PolgA*^*mut/mut*^ osteoclast culture at 2951 µm per osteoclast compared to 1737 µm of resorption on average by wild type osteoclasts (*p* = 0.0134).

High levels of variability are observed between dentine discs within individual cell lines and also between cell lines of the same genotype for number of pits and also in the resorption area recorded per osteoclast, particularly within the *PolgA*^*mut/mut*^ group.

### Respiratory chain protein expression declines in osteoblasts and osteoclasts of aging mice, and at an accelerated rate in *PolgA*^*mut/mut*^ mice

**Figure 6:**
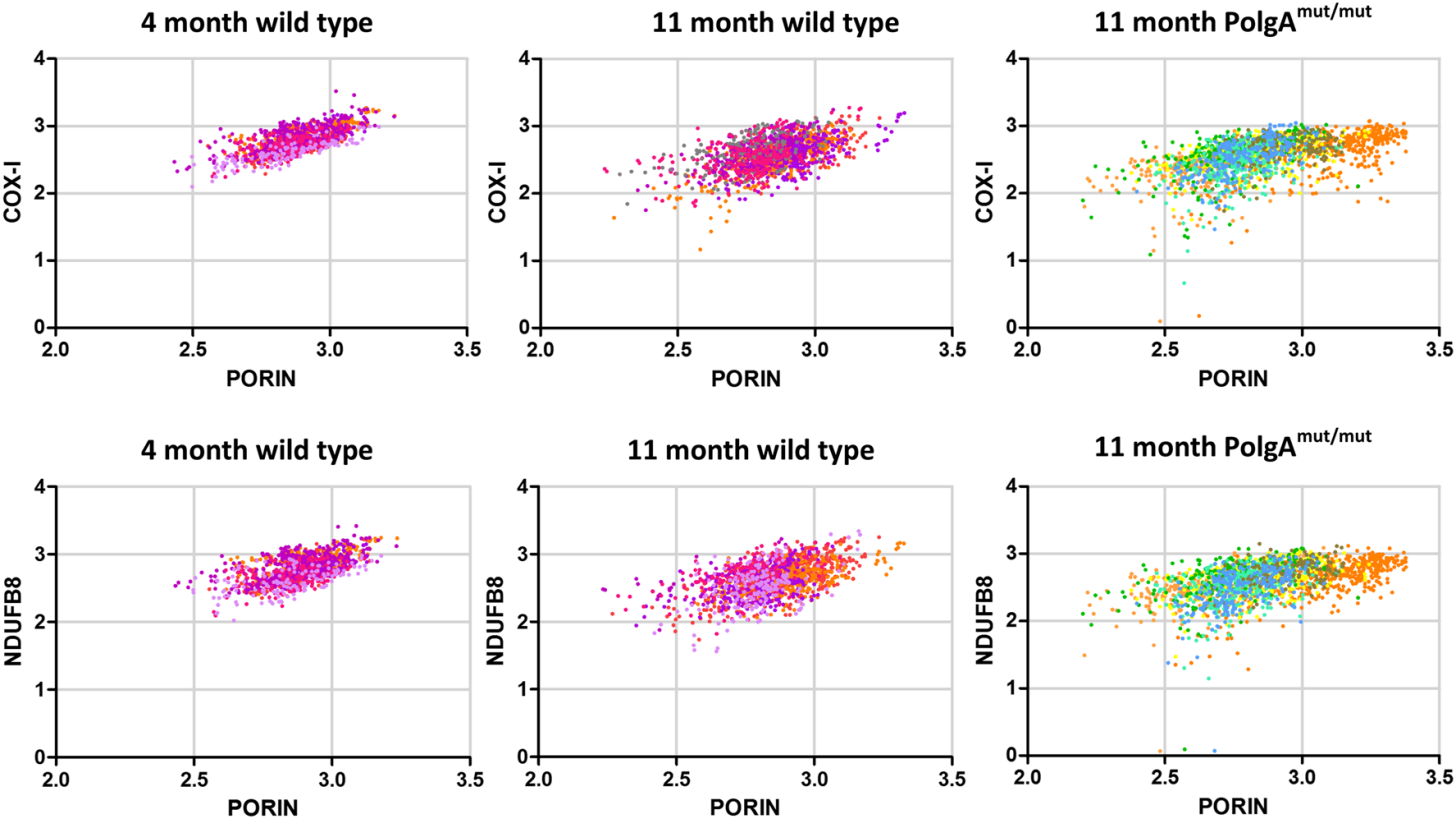
NDUFB8:Porin and COX-I:Porin in mouse osteoclasts. Log transformed background corrected signal intensities of porin, COX-I and NDUFB8 within individual osteoclasts were recorded. A linear association of NDUFB8 and COX-I to porin is present which is strongest in wild type mice aged 4 months. With increasing age in wild type mice, increasing variation in COX-I and NDUFB8 signal occurs with some loss of the linear relationship. This change is more marked in *PolgA*^*mut/mut*^ mice aged 11 months in which many cells exhibited low COX-I and NDUFB8 protein expression (different colours within each age group signify different cell lines).

**Figure 7:**
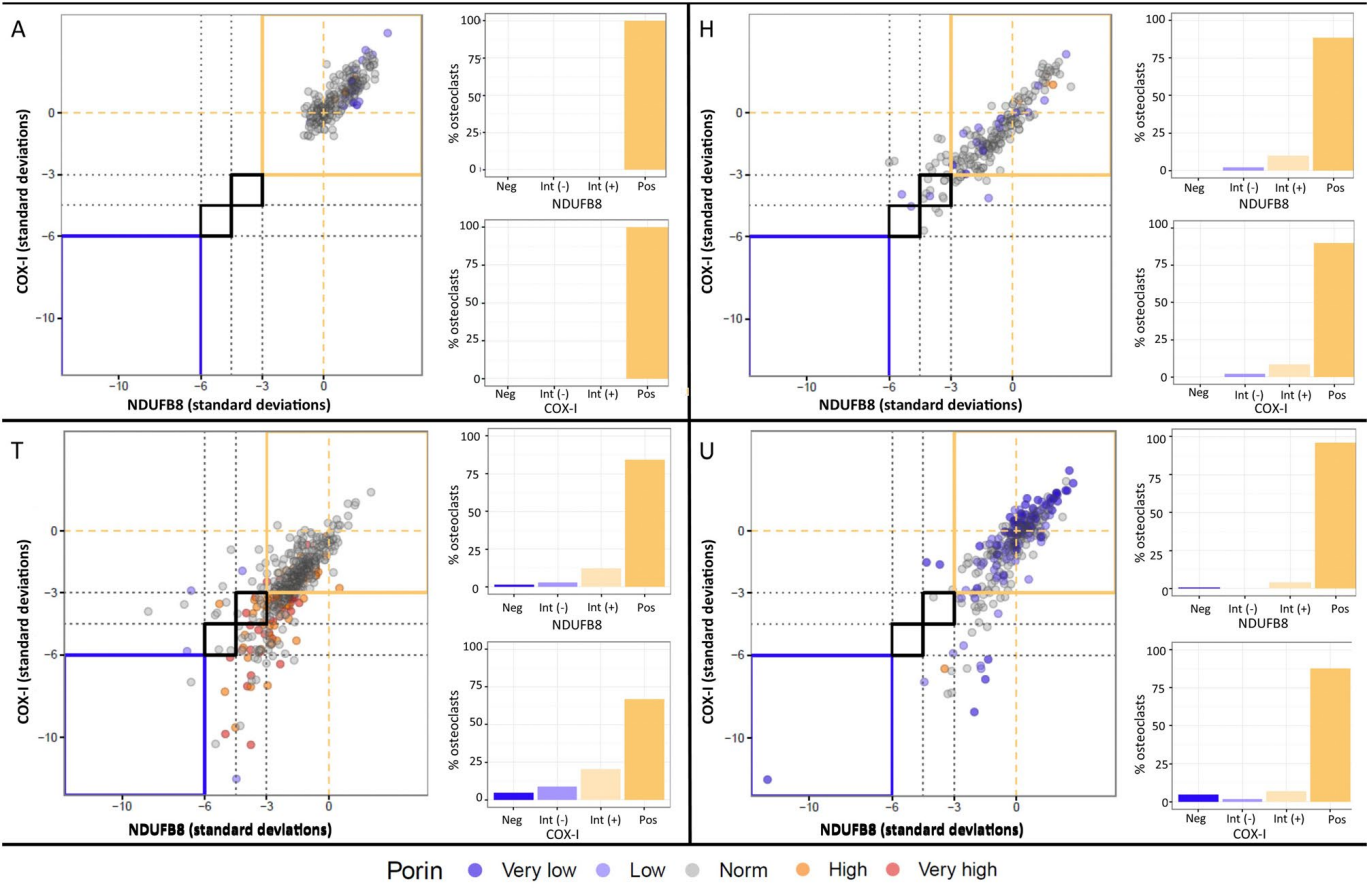
Representative graphs of Z-scored NDUFB8:Porin and COX-I:Porin for wild type and *PolgA*^*mut/mut*^ mice osteoclasts. Z-scores are derived for porin, NDUFB8 and COX-I from the mean and standard deviations of NDUFB8:porin and COX-I:porin relationships, in wild type controls aged 4 months. The Z scores for NDUFB8:porin and COX-I:porin are plotted against each other for each mouse, with each dot representing an individual osteoclast and it’s colour signifying the porin level (dark purple, very low; light purple, low; grey, normal; orange, high; red, very high). The typical appearances for a wild type mouse aged 4 months (**A**), wild type mouse aged 11 months (**H**), and *PolgA*^*mut/mut*^ mice aged 11 months (**T** and **U**) are shown (lettering corresponds to that in Supplemental Data S5 and S6). Most data points are no more than 3SD from the mean in the control group (wild type mice aged 4 months). However, in wild type mice aged 11 months reduced NDUFB8 and COX-I protein expression is observed and this deficit occurs to a greater degree in *PolgA*^*mut/mut*^ mice of the same age. The percentage of osteoclasts which are positive, intermediate positive, intermediate negative or negative for COX-I and NDUFB8 for each mouse are also shown, in the smaller panels to the right of each graph.

We have previously shown that a significant reduction in mitochondrial protein expression within osteoblasts occurs between the age of 4 and 11 months in wild type mice, with the levels of the respiratory chain proteins COX-I and NDUFB8 levels declining by 1.8 SD and 1.9 SD respectively. In *PolgA*^*mut/mut*^ mice aged 11 months, COX-I and NDUFB8 levels are 13.4SD and 8.2 SD lower respectively in osteoblasts when compared to osteoblasts within wild type mice aged 4 months^44^. Here we investigated mitochondrial protein expression within osteoclasts using the same methodology. Mitochondria within osteoclasts of wild type mice aged 4 months (n = 7) and 11 months (n = 7) were compared to 11 month *PolgA*^*mut/mut*^ mice (n = 7). The relationship between mitochondrial mass (porin), COX-I and NDUFB8 protein abundance in 4 month-old control mice is generally linear albeit with some variation, and there is evidence that some osteoclasts express low levels of COX-I and NDUFB8 even in these young control mice. In wild type mice aged 11 months, visibly higher variation was apparent with some loss of the linear relationship. This was even more apparent in 11 month *PolgA*^*mut/mut*^ animals with many cells observed displaying low COX-I and NDUFB8 protein abundance (Fig. 6). In osteoclasts from wild-type mice, mean COX-I and NDUFB8 levels decline with age by 0.76 SD and 0.36 SD respectively, from 4 to 11 months. In 11 month *PolgA*^*mut/mut*^ osteoclasts, COX-I and NDUFB8 protein expression in was 1.22 SD and 0.77 SD lower than in osteoclasts from wild type mice aged 4 months. When age-matched wild type and *PolgA*^*mut/mut*^ mice were compared (both 11 months old), COX-I and NDUFB8 levels in the latter were found to be 0.46 SD and 0.36SD lower respectively in *PolgA*^*mut/mut*^ osteoclasts. Numerical data for individual mice is shown in Supplemental Data S5. On average, 15.2% and 6.4% of *PolgA*^*mut/mut*^ osteoclasts showed some degree of COX-I and NDUFB8 deficiency respectively (intermediate positive, intermediate negative or negative classification), compared to 10.8% and 3.6% of osteoclasts from wild type mice aged 11 months. Corresponding individual graphs for all mice, depicting NDUFB8:porin and COX-I:porin ratios following Z-score analysis, which clearly show deficiency type and severity are provided in Supplemental Data S6. Figure 7 shows representative examples of these graphs for 4 month old wild type, 11 month old wild type and 11 month old *PolgA*^*mut/mut*^ mice.

## Discussion

With age, somatic mtDNA mutations accumulate in mitotic and post mitotic tissue, and somatic stem cell precursors^60^. We have demonstrated that osteoblasts^44^ osteoclast accumulate mitochondrial defects occur within ageing wild type mice and at an accelerated rate in *PolgA*^*mut/mut*^ mice, and that these findings correspond to increased bone loss, reduced bone formation rate, reduced osteoblast population density, and increased osteoclast population density in vivo*.* A corresponding decline in the capacity of osteogenic cells to form mineralised matrix, and increased osteoclast resorption activity in vitro were also demonstrated. We found that exercise did not affect bone mass or rescue the impaired function of extracted osteoblasts.

Mutations and consequent dysfunction in other tissues in the *PolgA*^*mut/mut*^ mouse model are cumulative with age^37^, hence our assessment of bone phenotype at different ages. With micro CT scanning, we found a significant reduction in lumbar vertebral BV/TV in male *PolgA*^*mut/mut*^ mice as young as 4 months and by 7 months in female *PolgA*^*mut/mut*^ mice. With advancing age, accelerated lumbar spine bone loss continues with significant deficits shown in male *PolgA*^*mut/mut*^ mice at 7 and 11 months, and female *PolgA*^*mut/mut*^ mice at 11 months, compared to age and sex matched wild type mice. Femoral trabecular bone shows a similar pattern of accelerated loss in *PolgA*^*mut/mut*^ mice with significant reductions in BV/TV seen in females by 7 months, and males by 11 months. It is not clear why the rate of femoral trabecular bone loss appears to slow in female *PolgA*^*mut/mut*^ mice after the age of 7 months, with no significant difference in BV/TV observed by the age of 11 months when compared with age matched wild type mice, although significant deficits in lumbar spine BV/TV were still apparent at this age. At each of the time points studied (4, 7 and 11 months), micro CT scan showed that BV/TV in male mice was higher than that of females, regardless of genotype, illustrating the significant role that sex hormone composition plays in bone biology. Our data for wild type mouse femoral and vertebral BV/TV is in keeping with previous work^61^ and the patterns of bone loss in *PolgA*^*mut/mut*^ mice mirrors that seen in wild type mice, further validating the usefulness of this model. In keeping with these findings, in vivo assessment showed significantly reduced BV/TV in *PolgA*^*mut/mut*^ mice compared to age and sex matched wild type mice.

Analysis of bone formation rates in vivo showed significantly reduced levels of osteogenesis at the mineralisation front in *PolgA*^*mut/mut*^ mice compared to age and sex matched wild type mice. This correlated with a significantly reduced population density of osteoblasts observed in this mouse model. Mitochondrial dysfunction in other tissues and organs may contribute to the osteoporotic phenotype of *PolgA*^*mut/mut*^ mice, hence our desire to study the function of osteogenic cells in vitro*,* with the effects of whole body physiology eradicated. In keeping with the reduced bone formation rates observed in vivo*, PolgA*^*mut/mut*^ ALP positive osteogenic cells produced significantly less mineralised matrix in vitro than age and sex matched wild type cells. Mineralised matrix was produced in abundance when using osteogenic cells derived from wild type bulk unfractionated skeletal mesenchymal cells in keeping with work using human and mouse MSCs^62,63^. However, with increasing age of wild type mice, which accumulate mitochondrial DNA mutations at a much slower rate than their *PolgA*^*mut/mut*^ counterparts, declining levels of mineralised matrix were produced, a finding which correlates with increasing in vivo bone loss and reducing osteoblast mitochondrial respiratory protein expression with advancing age^44^. A limitation in our study is the use of bulk unfractionated mesenchymal cells, rather than the use of purified osteogenic mesenchymal stem cells harvested using a method such as flow cytometry, and it would be useful to validate our findings using a method such as this.

MtDNA mutations are known to accumulate in stem cell populations with age^64^. The effect of these mtDNA mutations has been shown to vary depending on the cell line affected^65,66^. There have been no previous reports of the effects on cells of the MSC lineage. Although the population densities of ALP producing cells seen in our in vitro analysis showed no discernible difference between *PolgA*^*mut/mut*^ and wild type cell lines, our in vivo analysis showed significant reduction in numbers of mature bone lining osteoblasts. Clearly mitochondrial dysfunction is associated with abnormal differentiation of MSCs through an osteogenic lineage, with reduced population densities of mature bone lining osteoblasts occurring and impaired osteogenic function, as demonstrated by reduced in vivo bone formation rates. The capacity of osteogenic cells to produce mineralised matrix in vitro is also significantly impaired. During osteogenic differentiation of human MSCs, increasing levels of intracellular ATP content, mitochondrial DNA (mtDNA) copy number and protein subunits of respiratory enzymes occur, which correlates with increased oxidative phosphorylation during early differentiation^67,68^. *Wnt* signalling is vital for osteoblast differentiation, proliferation and subsequent mineralisation and it also appears to be intrinsically linked to mitochondrial biogenesis^69,70^. This relationship appears to be mutual as when mitochondrial biogenesis is enhanced following upregulation of TFAM activity in mouse mesenchymal cells in vitro, *Wnt* induced β-catenin expression and osteogenesis is significantly enhanced^69^.

Non-weight bearing states and weightlessness in space are known to cause bone loss in humans and animal models^71,72^. We found no significant difference in the weight of wild type and *PolgA*^*mut/mut*^ mice aged 4 and 7 months to account for differences in bone loss (data not shown), and Trifunovic et. al. did not find a significant difference in body weight until the age of 39 weeks (37). However, we acknowledge that significantly reduced body weight in *PolgA*^*mut/mut*^ mice by the age of 11 months may contribute to accelerated bone loss at this stage. Exercise has been shown to enhance respiratory chain activity in human muscle ^42^ and a similar exercise regime to ours has been shown to be protective against the ageing phenotype of *PolgA*^*mut/mut*^ mice when applied from the ages of 3 to 8 months, although the effects of exercise on bone loss was not evaluated^40^. We found that exercise did not affect bone mass in male *PolgA*^*mut/mut*^ mice and that osteogenic cells extracted from these mice exhibited the same reduced functional capacity as cells from non-exercised *PolgA*^*mut/mut*^ mice of the same age. Subtle variations between the exercise protocols used in ours and previous work may have contributed to the lack of effect observed. Recent work studying the effects of exercise on bone mass in wild type C57BL/6 mice shows that the intensity of exercise is critical with no significant increase in bone mass occurring when intensity is too low or too high^73^. The authors suggest that a minimum threshold of exercise must be reached to achieve a positive effect on bone density accrual and hypothesise that increased free radical production with excessive exercise, impairs bone mass accumulation.

Our analysis of osteoclast mitochondrial respiratory chain protein expression within osteoclasts showed that both COX-I and NDUFB8 protein expression levels decline with advancing age in wild type mouse osteoclasts and at an accelerated rate in *PolgA*^*mut/mut*^ mice. However, the decline was much more modest than that observed in osteoblasts ^44^. These results suggest that cells of the osteoclast lineage are less vulnerable to mitochondrial dysfunction with advancing age and in the *PolgA*^*mut/mut*^ mouse model, than cells of the osteoblast lineage. Alternatively, osteoclasts may be more dependent on preserved mitochondrial function, perhaps meaning that only those with normal respiratory chain function develop or survive in vivo*.* We found that the respiratory chain deficiency observed in osteoclasts is associated with an increased osteoclast population density in vivo in *PolgA*^*mut/mut*^ mice*.* Geurts et al*.* also noted increased osteoclast numbers in vivo in this mouse model^74^. Increased mitochondrial biogenesis and activity has been shown to occur during osteoclast differentiation, suggesting some degree of normal mitochondrial function is important to the process^75–77^. It is likely that a threshold of acceptable mitochondrial function exists, above which the cell can continue to function. Stromal and osteoblast expression of RANKL and M-CSF has been shown to increase with advancing age in mice, and when these cells are used in vitro to invoke osteoclastogenesis in osteoclast precursors, the effects are enhanced when using donor osteoblasts and stromal cells from older animals^78^. It is not clear whether upregulation of these factors is related to mitochondrial dysfunction, but a potential increase of their expression in *PolgA*^*mut/mut*^ mice, may explain the findings in our in vivo work. Similar findings are apparent in human whole bone and cultured marrow cells, with increasing expression of RANKL and reducing expression of OPG with advancing age. Overexpression of RANKL or under expression of OPG has been shown to lead to severe osteopenia^79,80^. The osteoclast progenitor pool, the expression of RANK (receptor to RANKL) and c-fms (receptor to M-CSF) have also been shown to increase with age^78,81,82^. Again, it not clear whether these changes are intrinsically linked to changes in cellular mitochondrial function.

Osteoclast-specific knock out of TFAM, which results in a reduction in mitochondrial gene expression and consequent mitochondrial dysfunction, has been shown to be associated with enhanced osteoclast resorption activity despite significantly reduced ATP content and accelerated apoptosis ^38^. In keeping with these findings, our in vitro work also demonstrates enhanced osteoclast activity, with significantly increased resorption by *PolgA*^*mut/mut*^ osteoclasts with an increased ratio of pits per osteoclast, and increased ratio of dentine resorbed per osteoclast. Variability observed between dentine discs within individual cell lines and also between cell lines of the same genotype for number of pits and also in the resorption area recorded per osteoclast, is likely to be accounted for by the cell culturing process, related to the harvest of variable numbers of monocytic cells when removing non-adherent cells from flasks.

Whilst osteoclast differentiation and resorption is clearly affected, the primary defect in bone homeostasis in the *PolgA*^*mut/mut*^ mouse model, appears to be one of reduced bone formation.

We believe that this is the first work to show that mitochondrial dysfunction, a universal feature of human ageing, severely impairs osteogenesis and is associated with accelerated bone loss. Although mitochondrial dysfunction in bone cells appears to have a direct effect on bone phenotype, the secondary effects on bone of mitochondrial dysfunction within other tissues, remains unclear. Further understanding of the potential role of mitochondrial dysfunction in failing bone biology is crucial. Although *PolgA*^*mut/mut*^ mice accumulate mtDNA mutations at 3–5 times of wild type mice, the resultant mitochondrial dysfunction is sufficient by 4 months of age to cause marked dysfunction with associated bone loss. Although humans accumulate mtDNA mutations at a slower rate than this, the lifespan of humans is such that the cumulative effects of these on bone homeostasis is a potentially significant factor in the development of osteoporosis and further investigation using human tissues and cell lines is warranted.

## Supplementary information

Supplementary Information 1.
