# Supplementary material for: Mitochondrial dysfunction impairs osteogenesis, increases osteoclast activity, and accelerates age related bone loss
Source: Sci Rep. 2020 Jul 15;10:11643. doi: 10.1038/s41598-020-68566-2 (PMC7363892; doi:10.1038/s41598-020-68566-2)
Supplement: Supplementary file 1 — Supplementary Information 1. [file 41598_2020_68566_MOESM1_ESM.docx]

**Supplemental data**

Mitochondrial dysfunction impairs osteogenesis, increases osteoclast activity, and accelerates age related bone loss.

Philip F. Dobson^1,2^, Ella P. Dennis^3^, Daniel Hipps^1,2^, Amy Reeve^1,2^, Alex Laude^4^, Carla Bradshaw^1,3^, Craig Stamp^1,3^, Anna Smith^1,3^, David J. Deehan^2,*^, Doug M. Turnbull^1,2,*^, Laura C. Greaves^1,3,*^

1. Wellcome Centre for Mitochondrial Research, Newcastle University, Newcastle upon Tyne, NE2 4HH, UK
2. Translational and Clinical Research Institute, Newcastle University, Newcastle upon Tyne, NE2 4HH, UK
3. Biosciences Institute, Newcastle University, Newcastle upon Tyne, NE2 4HH, UK
4. Bioimaging Unit, Medical School, FMS Professional Services, Newcastle University, Newcastle upon Tyne, NE2 4HH, UK

*These authors contributed equally to this work.

| Sex | Age (months) | Genotype  (n=) | Average trabecular  BV/TV % | p  value | Average  trabecular thickness  (µm) | p  value | Average trabecular separation  (µm) | p value | Average trabecular number  (per µm) | p  value | Cortical thickness  (µm) | p  value |
| --- | --- | --- | --- | --- | --- | --- | --- | --- | --- | --- | --- | --- |
| Male | 4 | Wild type  (8) | 21.7  (17.5 - 29.4) | 0.329 | 53.4  (47.2 - 61.2) | 0.663 | 165.8  (128.0 - 184.0) | 0.639 | 4.03E-03  (3.6E-03 - 4.8E-03) | 0.275 | 217.3  (193.0 - 240.0) | 0.077 |
|  |  | *PolgA^mut/mut^* (8) | 19.9  (16.9 - 22.9) |  | 52.7  (49.6 - 57.2) |  | 173.0  (123.3 - 198.3) |  | 3.78E-03  (3.2E-03 - 4.4E-02) |  | 204.2  (185.0 - 217.0) |  |
|  | 7 | Wild type  (6) | 13.4  (11.6 - 15.5) | 0.170 | 56.5  (42.9 - 63.8) | 0.454 | 236.0  (179.9 - 273.8) | 0.141 | 2.40E-03  (2.1E-03 - 2.9E-03) | 0.247 | 206.7  (187.0 - 226.0) | 0.500 |
|  |  | *PolgA^mut/mut^* (6) | 11.5  (6.8 - 14.3) |  | 53.3  (44.6 - 59.0) |  | 261.7  (238.7 - 288.0) |  | 2.14E-03  (1.5E-03 - 2.8E-03) |  | 197.5  (161.0 - 224.0) |  |
|  | 11 | Wild type  (8) | 12.3  (10.0 - 15.2) | <0.0001  *** | 60.6  (52.5 - 64.3) | 0.0009  *** | 267.6  (243.6 - 295.8) | 0.007  ** | 2.04E-03  (1.7E-03 - 2.4E-03) | 0.025  * | 227.3  (205.0 - 288.5) | 0.0008  *** |
|  |  | *PolgA^mut/mut^* (8) | 8.4  (6.5 - 10.6) |  | 51.2  (47.2 - 61.7 |  | 305.6  (272.4 - 340.6) |  | 1.65E-03  (1.2E-03 - 2.1E-03) |  | 184.7  (171.4 - 203.0) |  |
|  |  | Exercised *PolgA* (8) | 8.3  (5.4 - 14.0) | 0.907 (vs *PolgA*) | 50.1  (40.6 - 56.2) | 0.670 (vs *PolgA*) | 308.3  (197.4 - 392.13) | 0.921 (vs *PolgA*) | 1.70E-03  (1.0E-03 - 3.0E-03) | 0.874 (vs *PolgA*) | 172.0  (150.0 - 186.0) | 0.038 * (vs *PolgA* ) |
| Female | 4 | Wild type  (6) | 11.7  (8.8 - 17.0) | 0.254 | 53.5  (48.9 - 62.8) | 0.039  * | 246.4  (179.5 - 288.3) | 0.731 | 2.17-03  1.7E-03 - 2.3E-03 | 0.599 | 212.6  (209.0 - 218.0) | 0.323 |
|  |  | *PolgA^mut/mut^* (5) | 9.9  (8.1 - 12.7) |  | 48.0  (46.4 - 49.1) |  | 252.6  (231.0 - 275.1) |  | 2.06E-03  (1.7E-03 - 2.6E-03) |  | 208.8  (201.0 - 221.0) |  |
|  | 7 | Wild type  (5) | 8.53  (6.7 - 11.3) | 0.003  ** | 56.0  (51.5 - 61.9) | 0.251 | 317.7  (285.9 - 347.1) | <0.0001  *** | 1.53E-03  1.1E-03 - 2.0E-03 | 0.004  ** | 248.0  (238.0 - 259.0) | 0.032  * |
|  |  | *PolgA^mut/mut^* (5) | 4.2  (2.9 - 5.1) |  | 51.7  (43.8 - 59.0) |  | 392.9  (368.1 - 409.50) |  | 8.12E-04  (6.5E-04 - 9.6E-04) |  | 231.9  (220.0 - 248.3) |  |
|  | 11 | Wild type  (6) | 3.0  (2.4 - 3.3) | 0.029  * | 50.5  (42.6 - 55.2) | 0.572 | 453.4  (348.0 - 507.3) | 0.134 | 4.62E-03  (3.4E-04 - 5.9E-04) | 0.076 | 228.0  (196.0 - 248.0) | 0.005  ** |
|  |  | *PolgA^mut/mut^* (6) | 2.3  (1.5 - 3.0) |  | 49.0  (43.5 - 53.2) |  | 510.9  (450.3 - 626.7) |  | 5.92E-04  (4.7E-04 - 6.00E-04) |  | 192.9  (180.3 - 213.9) |  |

**(**BV – bone volume; TV – tissue volume; data ranges indicated in brackets)

**Supplemental Data S1. Femoral trabecular and cortical bone scan data**

Male and female *PolgA^mut/mut^* mice have significantly reduced trabecula bone density (bone volume/tissue volume; BV/TV) at 11 and 7 months respectively compared to age matched wild type littermates. Exercise was not associated with any difference in bone mass observed in 11 month old *PolgA^mut/mut^* mice. Significantly reduced levels of trabecular bone thickness, increased trabecular separation and reduced trabecular number in 11 month old *PolgA^mut/mut^* males, and significantly increased trabecular separation and reduced trabecular number in 7 month old *PolgA^mut/mut^* females are also observed. Female wild type mice demonstrate a significantly reduced trabecular bone density compared to age matched wild type males at all 3 ages studied (p < 0.001). Cortical thickness was also significantly reduced in male *PolgA^mut/mut^* mice at 11 months, and in female *PolgA^mut/mut^* at 7 and 11 months compared to age matched wild type littermates. (Unpaired 2 tailed t-tests.)

| Sex | Age (months) | Genotype  (n=) | Average trabecular  BV/TV % | p  value | Average  trabecular thickness  (µm) | p  value | Average trabecular separation  (µm) | p value | Average trabecular number  (per µm) | p  value |
| --- | --- | --- | --- | --- | --- | --- | --- | --- | --- | --- |
| Male | 4 | Wild type  (8) | 30.3  (27.9-33.3) | 0.035  * | 55.4  (53.2-58.7) | 0.060 | 158.3  (142.4-169.5) | 0.005  ** | 5.48E-03  (5.1E-03-5.9E-03) | 0.033  * |
|  |  | *PolgA^mut/mut^* (6) | 23.4  (12.0-30.6) |  | 47.1  (31.1-56.0) |  | 181.2  (156.3-200.1) |  | 4.84E-03  (3.8E-03-5.7E-03) |  |
|  | 7 | Wild type  (6) | 24.3  (21.3-27.2) | 0.033  * | 57.7  (51.0-64.7) | 0.016  * | 193.0  (178.0-219.0) | 0.027  * | 4.21E-03  (3.7E-03-4.7E-03) | 0.231 |
|  |  | *PolgA^mut/mut^* (6) | 19.8  (12.9-23.8) |  | 51.4  (46.2-55.4) |  | 222.3  (199.9-262.5) |  | 3.84E-03  (2.8E-03-4.4E-03) |  |
|  | 11 | Wild type  (15) | 25.0  (20.6-29.2) | <0.0001  *** | 55.02  (49.6-59.7) | 0.002  ** | 190.5  (160.0-222.4) | 0.003  ** | 4.56E-03  (3.8E-03-5.0E-03 | <0.0001  *** |
|  |  | PolgA*^mut/mut^* (10) | 15.4  (11.5-21.3) |  | 50.8  (44.4-57.2) |  | 251.9  (212.6-329.7) |  | 3.03E-03  (2.5E-03-3.9E-03) |  |
|  |  | Exercised *PolgA* (7) | 15.1  (8.0-21.8) | 0.877 (vs *PolgA*) | 46.7  (30.7-54.9) | 0.210 (vs *PolgA*) | 248.7  (180.62-364.6) | 0.898 (vs *PolgA*) | 3.21E-03  (2.0E-03-4.3E-03) | 0.579 (vs *PolgA*) |
| Female | 4 | Wild type  (5) | 24.9  (22.1-26.5) | 0.076 | 58.1  (53.2-61.4) | 0.222 | 235.8  (194.9-286.0) | 0.886 | 4.30E-03  (4.0E-03-4.8E-03) | 0.235 |
|  |  | *PolgA^mut/mut^* (5) | 22.8  (19.9-24.0) |  | 55.2  (49.9-57.5) |  | 238.2  (220.1-253.8) |  | 4.12E-03  (4.0E-03-4.3E-03) |  |
|  | 7 | Wild type  (7) | 26.1  (20.4-29.9) | 0.0004  *** | 59.5  (52.1-66.8) | 0.040  * | 244.3  (212.0-271.0) | 0.013  * | 4.37E-03  (3.9E-03-4.9E-03) | <0.0001  *** |
|  |  | *PolgA^mut/mut^* (7) | 17.8  (12.1-20.6) |  | 53.1  (43.0-56.7) |  | 273.3  (246.6-297.0 |  | 3.33E-03  (2.8E-03-3.7E-03) |  |
|  | 11 | Wild type  (7) | 20.2  (16.4-21.5) | 0.007  ** | 62.2  (57.0-67.2) | 0.0001  *** | 321.2  (287.9-393.2) | 0.232 | 3.25E-03  (2.8E-03-3.3E-03) | 0.122 |
|  |  | *PolgA^mut/mut^* (5) | 13.8  (11.0-21.3) |  | 50.2  (44.3-57.1) |  | 290.7  (228.0-329.3) |  | 2.72E-03  (2.2E-03-3.7E-03) |  |

**(**BV – bone volume; TV – tissue volume; data ranges indicated in brackets)

**Supplemental Data S2. Lumbar spine trabecular bone scan data**

Male *PolgA^mut/mut^* mice have significantly reduced lumbar spine trabecula bone density (bone volume/tissue volume; BV/TV) at 4, 7 and 11 months compared to age matched wild type littermates. Exercise was not associated with any difference in the bone mass observed in 11 month old *PolgA^mut/mut^* mice. Female *PolgA^mut/mut^* mice have significantly reduced lumbar spine trabecular bone density (BV/TV) at 7 and 11 months. Significantly reduced levels of trabecular bone thickness are seen in *PolgA^mut/mut^* male and female mice at 7 and 11 months. Significantly reduced trabecular separation is seen in male *PolgA^mut/mut^* mice at all ages and in *PolgA^mut/mut^* females at 7 months. Significantly reduced trabeculae are seen in male *PolgA^mut/mut^* mice at 4 and 11 months, and *PolgA^mut/mut^* females at 7 months. Female wild type mice demonstrate a significantly reduced trabecular bone density compared to age matched wild type males at 4 months and 11 months of age (p < 0.001) (Unpaired 2 tailed t-tests.)

| Age | Sex | genotype | Cell line | Alizarin red SA %  (26 images, 7 wells) | ALP  SA %  (26 images, 5 wells) | Alizarin red SA: ALP SA ratio | Avg. no. ALP positive cells/image | Avg. cell count/image (Hoescht) |
| --- | --- | --- | --- | --- | --- | --- | --- | --- |
| 4 months | Female | Wild type | 1 | 79.33 | 15.20 | 5.20 | 245 | 2238 |
|  |  |  | 2 | 83.41 | 10.33 | 8.07 | 163 | 228 |
|  |  |  | 3 | 61.92 | 7.01 | 8.83 | 106 | 845 |
|  |  |  | 4 | 58.44 | 7.84 | 7.45 | 125 | 930 |
|  |  |  | 5 | 58.3 | 7.69 | 7.58 | 123 | 1093 |
|  |  |  | 6 | 61.69 | 6.31 | 9.78 | 97 | 930 |
|  |  |  | 7 | 58.83 | 8.43 | 6.98 | 124 | 918 |
|  |  | *PolgA^mut/mut^* | 1 | 5.57 | 4.99 | 1.12 | 75 | 770 |
|  |  |  | 2 | 4.13 | 6.51 | 0.63 | 105 | 1113 |
|  |  |  | 3 | 4.92 | 5.98 | 0.82 | 88 | 1013 |
|  |  |  | 4 | 13.40 | 11.60 | 1.16 | 171 | 593 |
|  |  |  | 5 | 1.72 | 10.10 | 0.17 | 152 | 1905 |
|  |  |  | 6 | 72.86 | 23.65 | 3.08 | 356 | 635 |
|  | Male | Wild type | 1 | 55.50 | 7.25 | 7.66 | 108 | 508 |
|  |  |  | 2 | 80.48 | 11.12 | 7.23 | 173 | 555 |
|  |  |  | 3 | 56.21 | 8.08 | 6.96 | 132 | 858 |
|  |  |  | 4 | 72.73 | 13.16 | 5.53 | 208 | 668 |
|  |  |  | 5 | 73.58 | 7.83 | 9.40 | 120 | 653 |
|  |  |  | 6 | 67.29 | 11.07 | 6.08 | 165 | 1003 |
|  |  |  | 7 | 60.61 | 4.76 | 12.73 | 65 | 660 |
|  |  | *PolgA^mut/mut^* | 1 | 2.41 | 4.62 | 0.52 | 67 | 1003 |
|  |  |  | 2 | 3.77 | 7.69 | 0.49 | 118 | 1013 |
|  |  |  | 3 | 1.75 | 4.42 | 0.40 | 61 | 1013 |
|  |  |  | 4 | 1.57 | 2.57 | 0.61 | 44 | 2565 |
|  |  |  | 5 | 33.48 | 8.94 | 3.74 | 111 | 478 |
|  |  |  | 6 | 3.47 | 6.36 | 0.55 | 89 | 1105 |
|  |  |  | 7 | 2.90 | 5.30 | 0.55 | 77 | 1023 |
|  |  |  | 8 | 10.06 | 3.03 | 3.32 | 47 | 2163 |
| 7 months | Female | Wild type | 1 | 67.16 | 13.54 | 4.96 | 209 | 1435 |
|  |  |  | 2 | 49.08 | 12.85 | 3.82 | 186 | 1655 |
|  |  |  | 3 | 60.47 | 13.49 | 4.48 | 210 | 1915 |
|  |  |  | 4 | 58.18 | 16.65 | 3.49 | 264 | 1623 |
|  |  |  | 5 | 53.10 | 19.31 | 2.75 | 279 | 885 |
|  |  |  | 6 | 58.50 | 15.98 | 3.66 | 231 | 625 |
|  |  | *PolgA^mut/mut^* | 1 | 2.92 | 16.21 | 0.18 | 238 | 878 |
|  |  |  | 2 | 3.74 | 7.24 | 0.52 | 111 | 868 |
|  |  |  | 3 | 3.59 | 9.75 | 0.37 | 133 | 1308 |
|  |  |  | 4 | 2.84 | 9.97 | 0.24 | 149 | 1423 |
|  |  |  | 5 | 3.01 | 25.05 | 0.12 | 399 | 1505 |
|  |  |  | 6 | 1.56 | 4.93 | 0.31 | 74 | 890 |
|  | Male | Wild type | 1 | 69.70 | 14.67 | 4.75 | 228 | 1178 |
|  |  |  | 2 | 58.55 | 14.93 | 3.92 | 223 | 1045 |
|  |  |  | 3 | 60.71 | 14.99 | 4.05 | 233 | 945 |
|  |  |  | 4 | 61.48 | 14.37 | 4.28 | 218 | 1955 |
|  |  |  | 5 | 36.69 | 9.71 | 3.78 | 148 | 728 |
|  |  |  | 6 | 40.53 | 12.23 | 3.31 | 199 | 938 |
|  |  | *PolgA^mut/mut^* | 1 | 5.28 | 15.22 | 0.35 | 230 | 1305 |
|  |  |  | 2 | 11.44 | 13.95 | 0.82 | 215 | 695 |
|  |  |  | 3 | 0.41 | 1.93 | 0.21 | 30 | 528 |
|  |  |  | 4 | 0.83 | 13.15 | 0.06 | 185 | 748 |
|  |  |  | 5 | 1.19 | 11.74 | 0.1 | 176 | 503 |
|  |  |  | 6 | 1.66 | 24.43 | 0.07 | 350 | 1073 |

| Age | Sex | Genotype | Cell line | Alizarin red SA %  (26 images, 7 wells) | ALP SA %  (26 images, 5 wells) | Alizarin red SA: ALP SA ratio | Avg. no. ALP positive cells/image | Avg. cell count/image  (Hoescht) |
| --- | --- | --- | --- | --- | --- | --- | --- | --- |
| 11 months | Female | Wild type | 1 | 54.79 | 25.14 | 2.18 | 359 | 1988 |
|  |  |  | 2 | 54.56 | 25.43 | 2.15 | 382 | 1503 |
|  |  |  | 3 | 52.31 | 29.19 | 1.79 | 422 | 815 |
|  |  |  | 4 | 44.10 | 23.00 | 1.92 | 326 | 980 |
|  |  |  | 5 | 84.43 | 21.95 | 3.85 | 337 | 1278 |
|  |  |  | 6 | 90.31 | 15.38 | 5.87 | 246 | 888 |
|  |  |  | 7 | 90.52 | 23.61 | 3.83 | 367 | 970 |
|  |  |  | 8 | 88.67 | 28.82 | 3.08 | 430 | 1020 |
|  |  | *PolgA^mut/mut^* | 1 | 4.06 | 14.33 | 0.28 | 240 | 458 |
|  |  |  | 2 | 4.27 | 9.28 | 0.46 | 138 | 395 |
|  |  |  | 3 | 1.49 | 9.02 | 0.17 | 116 | 1350 |
|  |  |  | 4 | 2.07 | 13.03 | 0.16 | 201 | 950 |
|  |  |  | 5 | 17.89 | 12.74 | 1.40 | 188 | 368 |
|  | Male | Wild type | 1 | 74.40 | 27.36 | 2.72 | 408 | 1345 |
|  |  |  | 2 | 64.33 | 19.30 | 3.33 | 294 | 1215 |
|  |  |  | 3 | 76.09 | 15.98 | 4.76 | 244 | 1380 |
|  |  |  | 4 | 47.79 | 15.19 | 3.15 | 250 | 855 |
|  |  |  | 5 | 45.83 | 11.15 | 4.11 | 169 | 723 |
|  |  |  | 6 | 36.39 | 9.48 | 3.84 | 149 | 1928 |
|  |  | *PolgA^mut/mut^* | 1 | 1.46 | 8.02 | 0.18 | 114 | 768 |
|  |  |  | 2 | 2.16 | 8.39 | 0.26 | 134 | 333 |
|  |  |  | 3 | 5.01 | 9.42 | 0.53 | 129 | 725 |
|  |  |  | 4 | 1.60 | 4.60 | 0.35 | 68 | 350 |
|  |  |  | 5 | 1.67 | 10.66 | 0.16 | 145 | 1008 |
|  |  |  | 6 | 1.08 | 3.75 | 0.23 | 59 | 1225 |
|  |  |  | 7 | 3.54 | 5.94 | 0.60 | 84 | 825 |
|  |  |  | 8 | 1.90 | 10.54 | 0.18 | 164 | 893 |
|  |  | Exercised *PolgA^mut/mut^* | 1 | 5.10 | 34.28 | 0.15 | 477 | 615 |
|  |  |  | 2 | 1.46 | 3.70 | 0.39 | 53 | 453 |
|  |  |  | 3 | 1.28 | 8.92 | 0.14 | 134 | 1058 |
|  |  |  | 4 | 7.12 | 13.69 | 0.52 | 169 | 1080 |
|  |  |  | 5 | 2.63 | 5.04 | 0.52 | 77 | 338 |
|  |  |  | 6 | 3.30 | 7.66 | 0.43 | 118 | 1153 |
|  |  |  | 7 | 3.05 | 8.56 | 0.36 | 127 | 1075 |
|  |  |  | 8 | 1.17 | 5.41 | 0.22 | 74 | 428 |

**Supplemental Data S3. Average surface area of alizarin red and ALP staining, and cell counts for individual cell lines**

Surface areas (SA) for alizarin red staining (representing mineralised matrix formation) across 7 wells and SA of ALP positive cells across 5 wells, for individual cell lines are shown. Average total cell counts (Hoescht staining) from 15 images and average ALP positive cell counts from 26 images at 4x magnification (5 wells imaged) are also shown – nuclear staining is not specific to ALP positive cells and is used to derive the population density of ALP expressing osteogenic cells for each cell line.


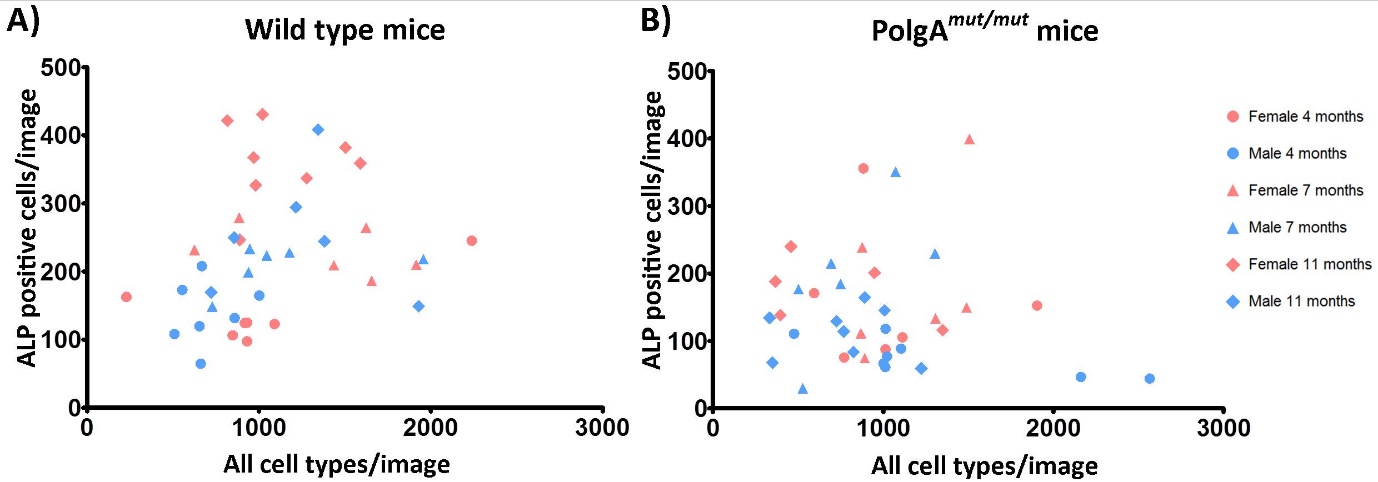


**Supplemental Data S4. Correlation of alkaline phosphatase (ALP) positive cells to total cell number**

Average number of osteogenic ALP positive cells was correlated with average number of all cell types, identified by Hoechst nuclear staining (5 wells per cell line). This was performed for wild type (A) and *PolgA^mut/mut^* (B) cells extracted from male and female mice aged 4, 7 and 11 months. No significant positive correlation (Pearson) is found in any cell line between the formation of ALP positive cells and cell number, highlighting the importance of correlating surface area of mineralised matrix formation directly to the surface area occupied by ALP expressing cells.

|  |  | **NDUFB8 osteoclasts** | | | | | **COX-I osteoclasts** | | | | |
| --- | --- | --- | --- | --- | --- | --- | --- | --- | --- | --- | --- |
|  | **Mouse** | **Pos** | **Int (+)** | **Int (-)** | **Neg** | **z-score** | **Pos** | **Int (+)** | **Int (-)** | **Neg** | **z-score** |
| 4 month wild type | A | 100.0% | 0.0% | 0.0% | 0.0% | 0.670997 | 100.0% | 0.0% | 0.0% | 0.0% | 0.46341 |
|  | B | 100.0% | 0.0% | 0.0% | 0.0% | -0.30878 | 100.0% | 0.0% | 0.0% | 0.0% | -0.1356 |
|  | C | 100.0% | 0.0% | 0.0% | 0.0% | 0.6936 | 100.0% | 0.0% | 0.0% | 0.0% | 0.76275 |
|  | D | 100.0% | 0.0% | 0.0% | 0.0% | -0.20711 | 100.0% | 0.0% | 0.0% | 0.0% | -0.04166 |
|  | E | 100.0% | 0.0% | 0.0% | 0.0% | -0.07022 | 100.0% | 0.0% | 0.0% | 0.0% | 0.01021 |
|  | F | 99.3% | 0.7% | 0.0% | 0.0% | -0.70274 | 100.0% | 0.0% | 0.0% | 0.0% | -0.82228 |
|  | G | 100.0% | 0.0% | 0.0% | 0.0% | -0.0221 | 100.0% | 0.0% | 0.0% | 0.0% | -0.10263 |
| 11 month wild type | H | 88.3% | 9.9% | 1.9% | 0.0% | -0.98045 | 89.7% | 8.5% | 1.9% | 0.0% | -1.11419 |
|  | I | 96.5% | 3.5% | 0.0% | 0.0% | 0.32396 | 93.0% | 7.0% | 0.0% | 0.0% | 0.05564 |
|  | J | 100.0% | 0.0% | 0.0% | 0.0% | 0.68333 | 93.6% | 2.1% | 4.2% | 0.0% | 0.84896 |
|  | K | 98.3% | 1.7% | 0.0% | 0.0% | -0.54610 | 95.4% | 4.6% | 0.0% | 0.0% | -0.44786 |
|  | L | 97.3% | 2.7% | 0.0% | 0.0% | -0.77915 | 79.5% | 16.8% | 3.7% | 0.0% | -1.59813 |
|  | M | 95.3% | 4.3% | 1.0% | 0.0% | -0.94677 | 83.7% | 14.0% | 0.7% | 1.7% | -1.73063 |
|  | N | 99.4% | 0.6% | 0.0% | 0.0% | -0.21290 | 89.3% | 9.5% | 1.2% | 0.0% | -1.21871 |
| 11 month *PolgA^mut/mut^* | O | 92.5% | 5.9% | 0.4% | 1.3% | -1.01973 | 89.1% | 6.7% | 3.8% | 0.4% | -0.98766 |
|  | P | 98.7% | 1.3% | 0.0% | 0.0% | -0.52358 | 88.7% | 7.5% | 2.5% | 1.3% | -1.12184 |
|  | Q | 91.4% | 6.4% | 1.6% | 0.6% | -0.83926 | 81.5% | 13.1% | 3.2% | 2.2% | -1.38334 |
|  | R | 96.5% | 1.8% | 1.3% | 0.4% | 0.08387 | 91.2% | 4.4% | 1.3% | 3.1% | `-0.32878 |
|  | S | 96.2% | 3.5% | 0.3% | 0.0% | -0.44115 | 88.9% | 8.2% | 1.7% | 1.2% | -0.96036 |
|  | T | 84.1% | 12.1% | 2.8% | 0.2% | -1.91283 | 66.6% | 20.0% | 8.6% | 4.7% | -2.71229 |
|  | U | 96.0% | 3.7% | 0.0% | 0.4% | -0.31307 | 87.5% | 6.6% | 1.5% | 4.4% | -0.95473 |

**Supplemental Data S5 Numerical data for individual mice used in osteoclast study, lettering corresponding to graphs in figure 7 and Supplemental Data S6**

Percentages of osteoclasts for individual mice which are positive, intermediate positive, intermediate negative and negative for NDUFB8 and COX-I protein expression are shown. Osteoblasts were classed as having normal levels (positive) if Z-scores were no more than -3SD from the mean, intermediate (+) if Z-scores were between -3SD and -4.5SD, intermediate (−) if Z-scores were between -4.5SD and -6SD, and deficient if Z-scores were more than -6 SD below the mean. Average Z-scores for NDUFB8:porin and COX-I:porin are also shown.

**Supplemental Data S6. Z-score graphs for individual mice used in osteoclast quadruple immunofluorescence assay**
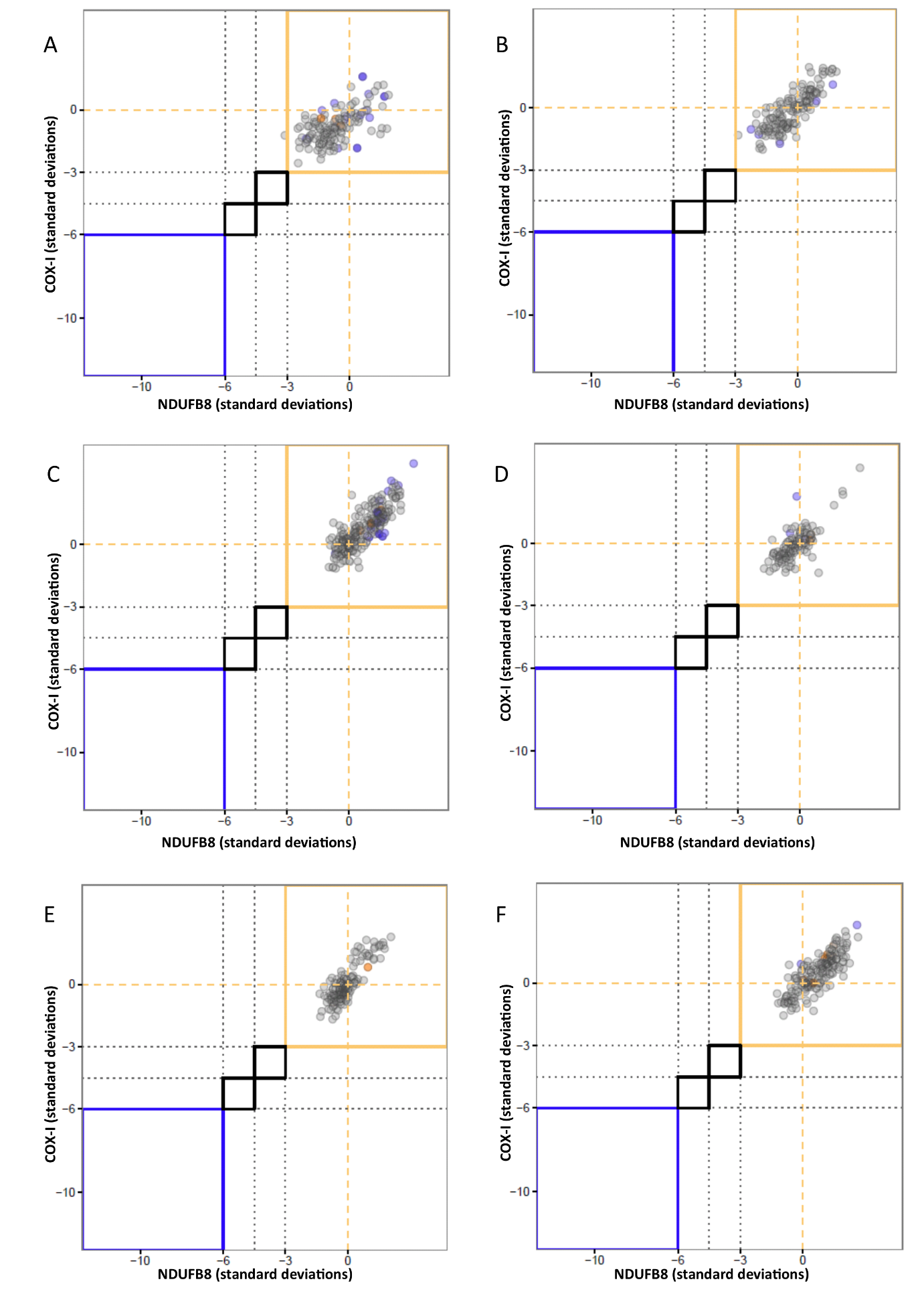

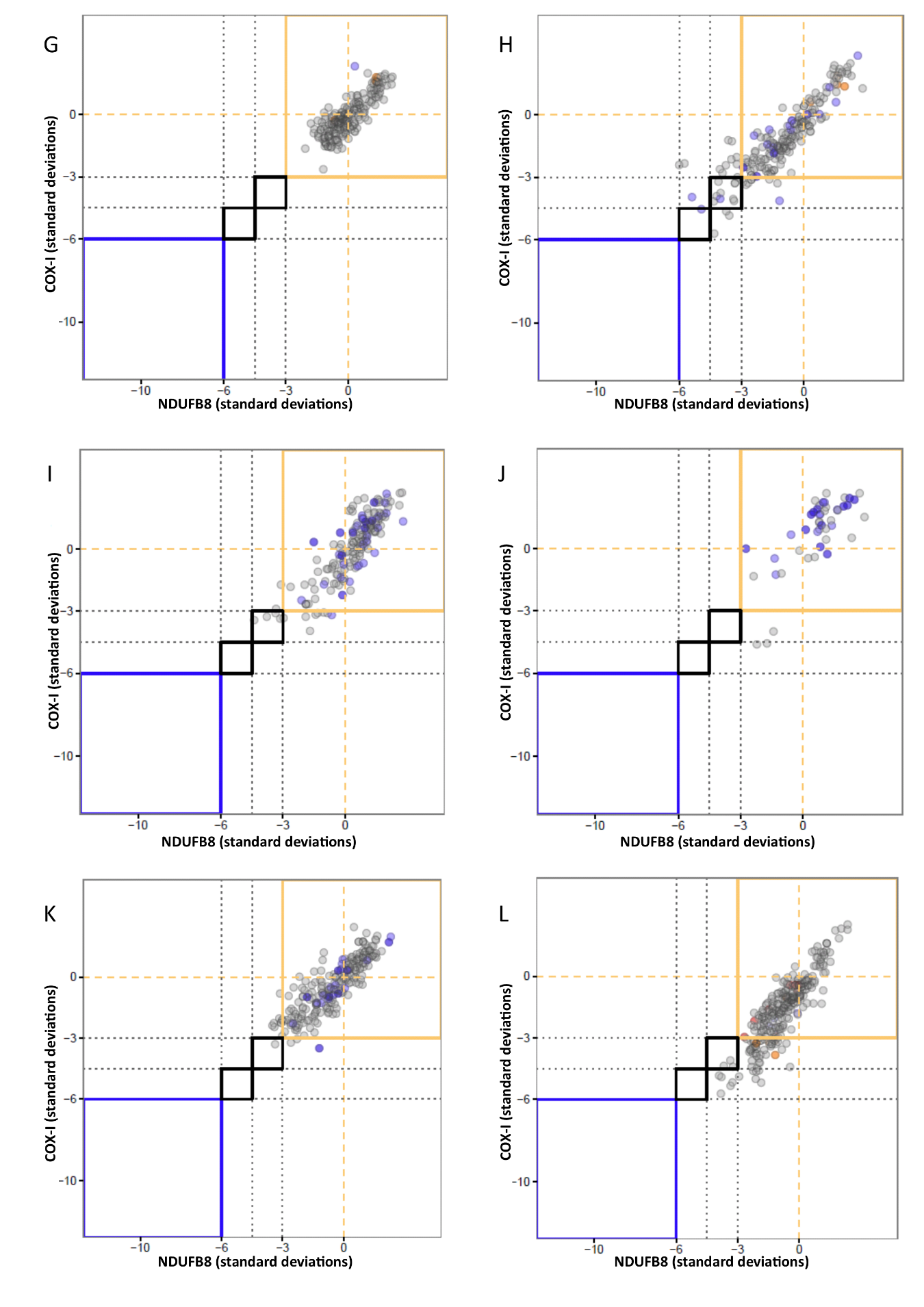


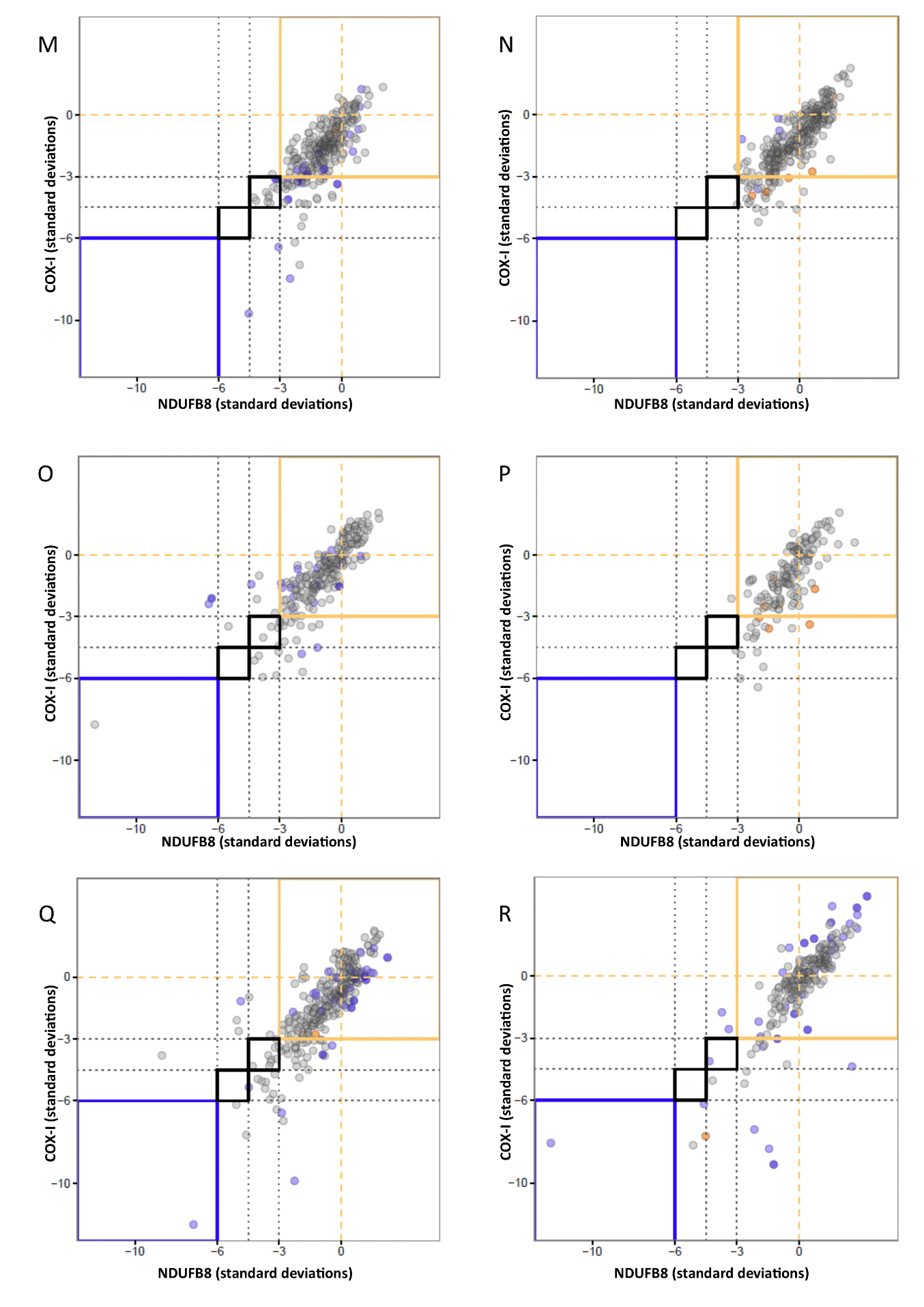


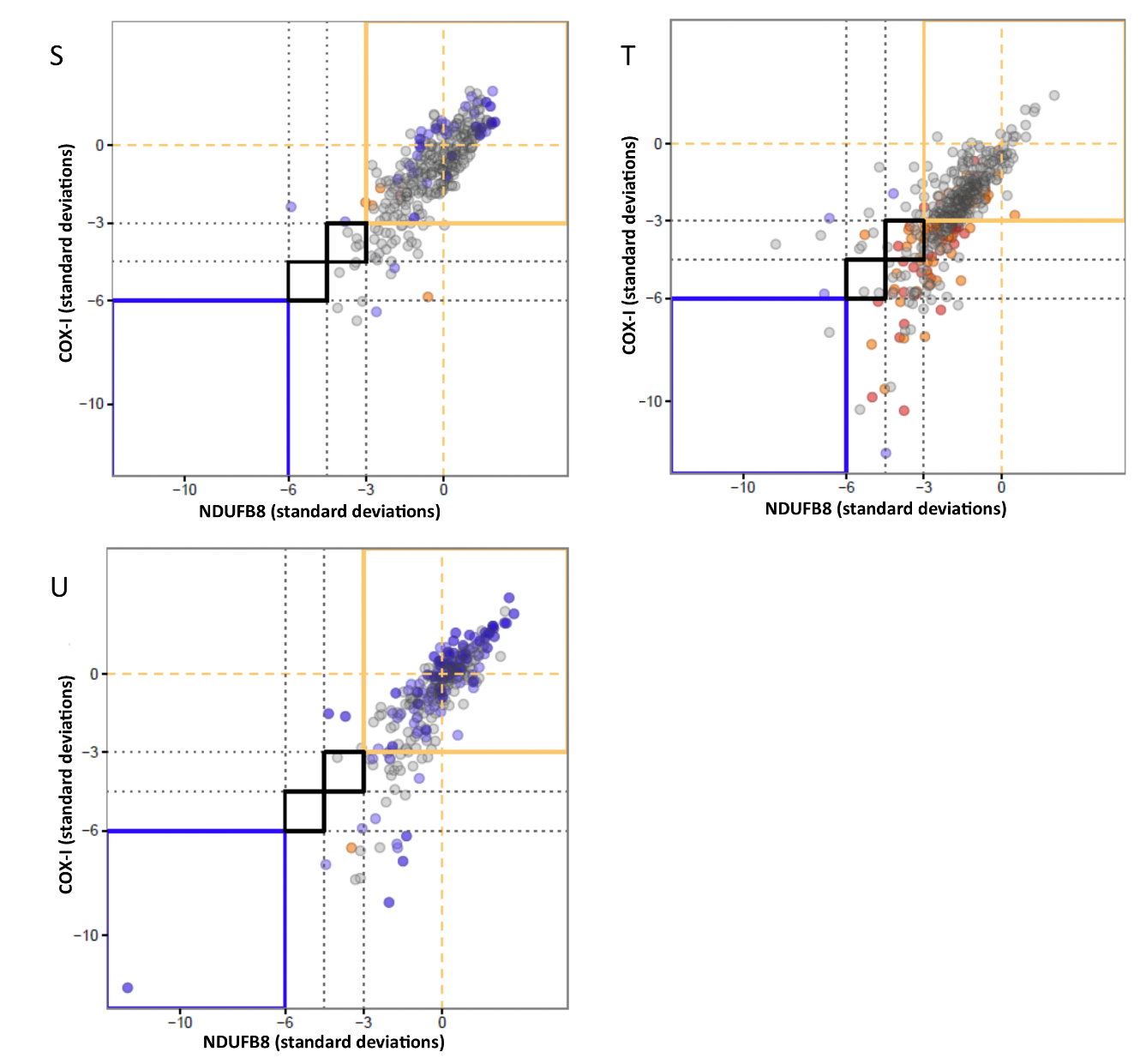


**Supplemental Data S6. Z-score NDUFB8:Porin and COX-I:Porin graphs for individual mice in osteoclast quadruple immunofluorescence study.**

The mean and standard deviations of these relationships in the 4 month wild type controls are established to derive Z–scores for porin, NDUFB8 and COX-I. Z-score for NDUFB8:porin and COX-I:porin are plotted against each other for individual mice. Each dot represents a single osteoblast, colour coded by the porin level (dark purple, very low; light purple, low; beige, normal; orange, high; red, very high). A-G represents data from 4 month old wild type animals where the vast majority of data points are no more than 3SD from the mean of this young control group. H-N shows 11 month wild type animals and O-U represents 11 month *PolgA^mut/mut^* animals with increasing deviation from the young controls, demonstrating increasing NDUFB8 and COX-I deficiencies.
